# Goal directed therapy for suspected acute bacterial meningitis in adults and adolescents in sub-Saharan Africa

**DOI:** 10.1371/journal.pone.0186687

**Published:** 2017-10-27

**Authors:** Emma C. Wall, Mavuto Mukaka, Brigitte Denis, Veronica S. Mlozowa, Malango Msukwa, Khumbo Kasambala, Mulinda Nyrienda, Theresa J. Allain, Brian Faragher, Robert S. Heyderman, David G. Lalloo

**Affiliations:** 1 Malawi-Liverpool-Wellcome Trust Clinical Research Programme, Blantyre, Malawi; 2 Liverpool School of Tropical Medicine, Pembroke Place, Liverpool, United Kingdom; 3 Division of Infection and Immunity, University College London, London, United Kingdom; 4 Mahidol Oxford Tropical Medicine Research Unit (MORU), Bangkok, Thailand; 5 Oxford Centre for Tropical Medicine and Global Health, Nuffield Department of Medicine Research Building, University of Oxford, Oxford, United Kingdom; 6 Adult Emergency and Trauma Centre, Ministry of Health, Queen Elizabeth Central Hospital, Blantyre, Malawi; 7 Department of Medicine, College of Medicine, Blantyre, Malawi; Public Health England, UNITED KINGDOM

## Abstract

**Background:**

Mortality from acute bacterial meningitis (ABM) in sub-Saharan African adults and adolescents exceeds 50%. We tested if Goal Directed Therapy (GDT) was feasible for adults and adolescents with clinically suspected ABM in Malawi.

**Materials and methods:**

Sequential patient cohorts of adults and adolescents with clinically suspected ABM were recruited in the emergency department of a teaching hospital in Malawi using a before/after design. Routine care was monitored in year one (P1). In year two (P2), nurses delivered protocolised GDT (rapid antibiotics, airway support, oxygenation, seizure control and fluid resuscitation) to a second cohort. The primary endpoint was composite mean number of clinical goals attained. Secondary endpoints were individual goals attained and death or disability from proven or probable ABM at day 40.

**Results:**

563 patients with suspected ABM were enrolled in the study; 273 were monitored in P1; 290 patients with suspected ABM received GDT in P2. 61% were male, median age 33 years and 90% were HIV co-infected. ABM was proven or probable in 132 (23%) patients. GDT attained more clinical goals compared to routine care: composite mean number of goals in P1 was 0·55 vs. 1·57 in P2 GDT (p<0·001); Death or disability by day 40 from proven or probable ABM occurred in 29/57 (51%) in P1 and 38/60 (63%) in P2 (p = 0·19).

**Conclusion:**

Nurse-led GDT in a resource-constrained setting was associated with improved delivery of protocolised care. Outcome was unaffected.

**Trial registration:**

**www.isrctn.com** ISRCTN96218197

## Introduction

Worldwide, acute bacterial meningitis (ABM) in adults and adolescents is associated with substantial mortality and morbidity [1–4]. Prompt treatment of suspected ABM with parenteral antibiotics is routinely recommended and is associated with improvements in outcome in Europe and the United States [5–10]. The highest incidence of ABM with the poorest outcomes occurs in sub-Saharan Africa (SSA), [11, 12] 50–70% of adults with ABM die compared to 20–30% in better resourced settings [2, 13, 14]. Interventions including dexamethasone and glycerol have shown benefit in richer countries, but have failed to improve outcomes from ABM in Africa [14–17].

Improvements in outcome from sepsis using protocolised, goal directed therapy (GDT) have been reported [18, 19], leading to internationally accepted sepsis guidelines [20, 21]. GDT as an approach for management of suspected ABM has not been tested, little data exists from studies of GDT for life-threatening infection in resource-limited environments [22, 23].

We postulated that GDT might be beneficial for adult patients with ABM, by effectively targeting resources in this constrained setting to reduce in-hospital delay and optimise clinical management. We conducted a study of a resource-appropriate, clinical care bundle for the management of suspected ABM in adults and adolescents at a large teaching hospital in Blantyre, Malawi. The primary endpoint was the composite mean number of clinical goals achieved. Secondary endpoints were proportions of individual goals achieved; and death or disability from proven or probable ABM at day 40.

## Methods

### Patients

Adults and adolescents over the age of 14 years were screened at triage in the adult emergency and trauma centre (AETC) at Queen Elizabeth Central Hospital, Blantyre, Malawi and referred directly to the study team if the inclusion criteria for suspected ABM were met: temperature >38°C or <35.5°C, with one or more clinical features of meningitis: severe headache, neck stiffness, photophobia, confusion, coma or seizures [24]. Exclusion criteria were age <14 years, known terminal illness, alternative source of infection, primary sepsis without meningism or severe head injury.

All patients were assessed by an AETC physician, underwent lumbar puncture if no contra-indications existed [25] and received antibiotics (IV ceftriaxone 2g BD). ABM was defined as proven (positive CSF Gram’s stain, culture or retrospective PCR for causative organisms, irrespective of other CSF findings), or probable (negative microbiology with acute history and CSF pleocytosis of >50 cells/mm^3^ or clumped cells with >50% neutrophils or >50% lymphocytes with prior antibiotics and biochemical evidence of meningitis, CSF: Blood glucose ratio of <0.4, raised CSF protein >0.5g/L) [25].

ABM was excluded at 72 hours if CSF was culture negative without evidence of inflammation, CSF testing for cryptococcal (culture and or CrAg) was positive, or a clinical diagnosis of TB meningitis was assigned. Neither Gene-Expert Rif-TB or culture for TB were available. Shock was defined as per the 2008 surviving sepsis guidelines (tachycardia >100 bpm, mean arterial blood pressure <70mmHg, systolic blood pressure <90mmHg, blood lactate >4 mmol/L, capillary refill time of >2 seconds, skin mottling) [26].

All patients gave verbal assent for initial inclusion [27]; patients with proven or probable ABM gave written informed consent. If patients were not able to provide either assent or consent, a nominated patient guardian gave consent.

### Study design

We recruited two sequential cohorts of adults and adolescents with suspected ABM on admission to hospital over two years in a before/after design. Start and finish times were planned to ensure seasonal meningitis peaks were captured in each cohort. During the first phase (P1, January 2012-October 2012), the study team observed routine clinical care delivered in the first six hours and identified potential therapeutic goals, no additional intervention was made. In the second phase (P2, December 2012-October 2013), study nurses identified potential therapeutic goals and delivered a targeted care bundle over six hours. Delivery of the interventions in line with the goals identified (by routine care in P1 and then protocolised care in P2) was monitored over six-hours; all patients were transferred to ward-based care. The minimum clinical goal expected for all patients was delivery of parenteral antibiotics within one hour of admission. Data collection and non-care bundle procedures were identical in both phases. Randomisation and blinding were not done [28, 29]. Formal sample size calculations were not done for this non-randomised feasibility study, clinical efficacy was a secondary endpoint, no data exist on which to base effect size estimates of GDT for ABM. Based on previous incidence data, the study aimed to recruit 100 patients with proven/probable ABM to each cohort.

### Follow up

All enrolled patients were followed up for 48 hours. Patients with proven or probable ABM were followed up daily to in-hospital death or discharge, then contacted weekly by telephone until clinical review at day 40. Patients with an alternative diagnosis to bacterial meningitis were excluded at 72 hours and not followed up. Travel expenses were reimbursed for follow up to a value of MK 500 (2012 exchange rate: USD 2).

### Intervention

The clinical care bundle intervention was based on the UK Resuscitation Council’s guidelines for emergency interventions 2010, UK bacterial meningitis guidelines 2005, the Surviving Sepsis guidelines 2008, and 2006 sub-arachnoid haemorrhage guidance [7, 26, 30, 31]. All components were adapted for resource-limited environments. Where possible, the clinical care bundle was designed to correct physiological abnormalities associated with poor outcome from either ABM or sepsis [1, 2, 32–34]. The care bundle was delivered over a six hour time period, and comprised the following clinical goals: clinical review and delivery of parenteral ceftriaxone within one hour of registration, airway support with nasopharyngeal airway if GCS <8, head tilt to 30 degrees if GCS <11, oxygen via nasal cannulae if saturations <94%, fluid resuscitation as per 2008 surviving sepsis guidelines with Ringer’s lactate if clinical features of shock present, transfusion with packed red cells if haemoglobin <60mg/dL, control of acute seizure activity with parenteral diazepam or phenobarbitone, and correction of hypoglycaemia with oral or parenteral dextrose if blood glucose <4.0mmol/L.

### Study objectives, endpoints and adverse events

The primary objective of the study was to assess the feasibility of GDT for suspected ABM in a single hospital in SSA. The secondary objective was to determine if any effect on clinical outcome was observed in a sub-set of patients with proven or probable ABM. To assess the primary objective, the number of goals identified and achieved were recorded for each patient with suspected ABM. The primary endpoint was the composite mean number of goals achieved at six hours in patients with suspected ABM. This was determined as follows: in each cohort patients were grouped by numbers of potential goals identified; proportions of goals achieved to those identified were calculated. Weighted means of the total number of goals identified and achieved were formulated; composite mean numbers of goals achieved at six hours were derived.

Secondary endpoints were: proportions of individual goals achieved in patients with suspected ABM and death or disability (defined by ≥2 points on the modified Rankin score, mRS [35]) at day 40 in patients with proven or probable ABM. *A priori* planned sub-group analyses in patients with proven or probable ABM were also undertaken.

Monthly aggregated data, including unexpected adverse events, were sent to the chief investigators, two independent study physician-observers and the study statistician for safety monitoring. If any of these bodies determined concerns regarding increasing harm to study participants compared to historical data, a meeting to determine if the study should be stopped or paused was to be called.

### HIV testing

All patients were offered testing for HIV following national guidelines, surviving patients were counselled and tested before discharge. When HIV testing kits were not available, discharged patients were strongly advised to seek community testing, pseudo-anonymous testing using study numbers was done on retained samples at the end of the study. Patients newly diagnosed with HIV infection were started on co-trimoxazole prophylaxis and referred according to the national guidelines.

### Laboratory methods

CSF analyses were done at the externally quality assured MLW Laboratory [36]. CSF was cultured on blood and chocolate agar for 48 hours at 37°C; subculture, identification and antibiotic sensitivities were done according to BSAC guidelines [37]. Aerobic blood culture samples were cultured in the BacTec^®^ system for 5 days. CSF biochemistry was determined using the Beckman-Coulter^®^ analyser. Bacterial DNA was extracted as previously described [38] multiplex real-time PCR for *Haemophilus influenzae*, *Streptococcus pneumoniae* and *Neisseria meningitidis* was done using Fast Track Diagnostics kits (FTD, Netherlands). Point of care testing included blood glucose (Accu-chek, Aviva diagnostics), CSF cryptococcal antigen lateral flow assay (IMMY, New York, USA), haemoglobin (Hemocue), HIV antibodies (BioRad® Genie HIV 1&2), (Determine™ HIV 1&2 Abbott) and CSF/blood lactate (Lactate-pro®, Habidirect).

### Statistical analysis and data management

Data were entered in parallel onto paper case record forms and digitally using the REDCap data system (REDCap, Vanderbilt University, USA). Data were exported from REDCap into SPSS statistics version 20 (IBM statistics USA). Graphs were generated using GraphPad Prism version 6.

Outcomes were analysed on an intention to treat basis. All statistical tests were two tailed, p <0.05 determined statistical significance. 95% confidence intervals are presented for Odds and Rate Ratios. Comparisons of proportions of goal identified and achieved were calculated using Fisher’s exact test and logistic regression. Composite means were calculated using Poission regression.

Kaplan-Meier survival plots were performed to compare survival curves between the observational group and the goal directed therapy group. Statistical comparison was done using the non-parametric Wilcoxon Log rank test. Logistic regression was used to model associations between clinical outcomes and risk factors while controlling for confounding.

### Ethics

Institutional review board ethical approval was obtained both from the College of Medicine Research and Ethics Committee of the University of Malawi (P.01/10/980, January 2011), and the Liverpool School of Tropical Medicine Research Ethics Committee, UK (P10.70, November 2010). A protocol amendment was approved by both committees, dated December 2011, lowering the age of recruitment from 18 years to 14 years, to facilitate the inclusion of adolescents with meningitis.

The full study protocol is available dx.doi.org/10.17504/protocols.io.j5kcq4w

(S1 File), S2 File TREND checklist for trial reporting.

## Results

### Patient demographics and clinical features

**Fig 1:**
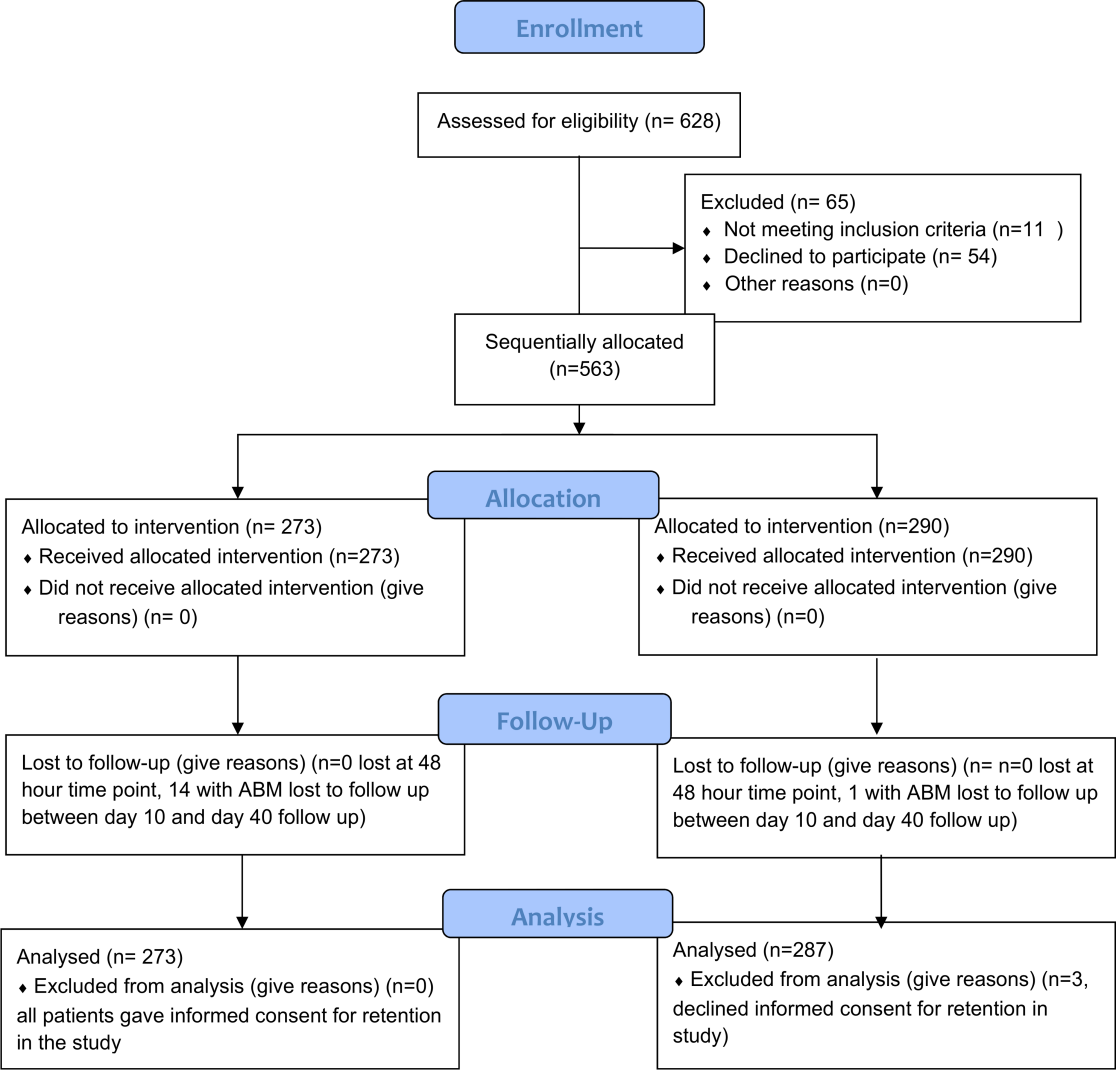
Enrolment of all participants with suspected bacterial meningitis.

Between January 2012 and October 2013, 628 patients were screened across both cohorts (Fig 1). Eleven patients did not meet screening inclusion criteria and were not included, a further 54 patients meeting the screening inclusion criteria declined verbal assent for inclusion in the study. Five hundred and sixty-three patients with suspected ABM were included and followed for 72 hours across the cohorts (Fig 1); 273 patients in P1 and 290 patients in P2.

### Suspected ABM

Median age of patients with suspected ABM was 32 years (range 14–82) in P1 and 35 years (14–90) in P2, 48% and 40% respectively were female (Table 1). Rates of HIV co-infection where test results were available were high; 134/146 (91%) in P1 and 146/165 (88%) in P2. In patients with known HIV co-infection, 102/134 (76%) and 96/146 (66%) respectively were taking antiretroviral therapy (ART).

**Table 1:** Baseline characteristics of all screened study participants with suspected bacterial meningitis.

Characteristic	Phase 1 N = 273	Phase 2 N = 290	P-value*
Female (no. %)	130 (48)	117/290 (40)	0.08
Age - years (Median, range)	32 (14-82)	35 (14–90)	0.02
HIV co-infection (no. %)	134/146 (91)	146/165 (88)	0.46
WHO HIV stage 3/4 (no. %)	166/267 (62)	148/288 (51)	0.01
Antiretroviral therapy (no. %)	102/134 (76)	96/146 (66)	0.11
Headache (no. %)	242/273 (88.6)	261/290 (90)	0.68
Admission Seizures (no. %)	72/273 (26)	61/290 (21)	0.13
Neck stiffness (no. %)	161/272 (59)	138/290 (48)	0.007
Photophobia (no. %)	58/273 (21)	91/290 (31)	0.01
Cranial nerve palsy (no. %)	1/259 (0.4)	11/290 (4)	0.007
Moribund on admission (no. %)†	25/269 (9)	15/290 (5)	0.07
Clinical evidence of pneumonia (no. %)	24/271 (9)	40/290 (14)	0.08
Focal neurology (no. %)	20/270 (7)	26/289 (9)	0.54
Pre-illness modified Rankin Score ≥2 (no. %)	26/271 (9.5)	24/290 (8)	0.65
Acute seizures (no. %)	13/237 (5.5)	46/282 (16)	<0.001
Focal neurology (no. %)	20/270 (7)	26/289 (9)	0.54

*Univariate significance of differences between two phases

**†**expected to die within 24 hours of admission on hospital due to catastrophic presenting clinical features

No marked differences in clinical presentation between the two cohorts were observed in patients with suspected ABM (Table 1, S1 Table). In P2, patients were more likely to be older, have higher rates of acute seizures, cranial nerve palsies and photophobia, and have a WHO stage 3 or 4 defining condition. (S1 Table). The commonest clinical diagnoses in patients with suspected ABM were proven/probable ABM, bacterial sepsis, cryptococcal meningitis and TB meningitis (S2 Table).

### Proven or probable ABM

135 participants (24%) met CSF inclusion criteria for proven or probable ABM, 71 in P1 and 61 in P2, three patients declined informed consent and were excluded, 132 were followed up. 113 had a bacterial pathogen proven on laboratory testing (S3 Table), 22 additional participants were defined as probable ABM. The characteristics of proven/probable cases were similar (S4 Table), Malawi Adult Meningitis Scores (MAMS) [39] were similar, median MAMS = 157 (95% CI 132–182) in P1 and 149 (110–190) p = 0.61.

### Attainment of clinical targets in patients with suspected ABM

The achievement of goals in the two cohorts of patients with suspected ABM is shown in Table 2. The composite mean number of goals identified was the same across the cohorts: P1 2.59 (SE 0.148), in P2 2.52 (0.177), RR 1.027 (95% CI 0.85–1.23), p = 0.77. GDT substantially increased the composite mean number of identified goals achieved in patients with suspected ABM from 0.55 (SE. 0.10) in P1 to 1.57 (0.12) in P2, rate ratio (RR) 2.39 (95% CI 2.3–5.1) (p<0.001).

**Table 2:** Achievement of goals in patients with suspected bacterial meningitis.

	Phase 1 n = 273	Phase 2 n = 290	Rate ratio (95% CI)	p-value
Goals identified (Mean, s.e.)	2.59 (0.148)	2.52 (0.177)	1.03 (0.86, 1.23)	0.773
Goals achieved (Mean, s.e)	0.55 (0.102)	1.57 (0.128)	2.4 (2.3, 5.1)	<0.001
Goals identified per patient (no.)	Phase 1 Goals achieved / no. identified (%)	Phase 2 Goals achieved no. (%)	Odds ratio (95% CI)	p-value
1	12/74 (16)	20/65 (30)	2.4 (1.1, 5.6)	0.031
2	18/114 (16)	52/126 (41)	5.8 (2.3,14.7)	<0.001
3	5/49 (10)	36/50 (72)	36.1 (3.6,354)	0.002
4	0/26 (0)	7/30 (23)	NA	-
5	0/7 (0)	4/12 (33)	NA	-
6	0/2 (0)	0/6 (0)	NA	-
7	0/1 (0)	0/1 (0)	NA	-

The most frequent number of goals identified was two (114 patients in P1 and 126 patients in P2) and greater than three goals identified was unusual (Table 2). As the number of goals identified increased, greater proportions of goals were achieved in P2 GDT cohort compared to P1.

GDT led to significant improvements in time to administration of antibiotics, airway insertion, head tilts and the use of oxygen (Table 3). Time from admission to clinical review was the same in both cohorts (median 35 minutes range 15–120 minutes), patients in P2 were significantly more likely to receive parenteral antibiotics within one hour (OR 9.6 95% CI 5.5: 16.9 p<0.001). GDT significantly increased the volumes of parenteral fluid administered (median volume in P1 = 750ml (IQR 500–750), increased to 1500ml (900-2200mls) in P2 (p = <0.001).

**Table 3:** Attainment of individual goals in patients with suspected bacterial meningitis.

Goal	Phase 1 Goals achieved no. (%)	Phase 2 Goals achieved no. (%)	p- value
**Airway placement if patient unconscious**	0/26 (0%)	14/20 (70%)	<0.001
**Head tilt if patient semi-conscious or unconscious**	0/41 (0%)	52/52 (100%)	<0.001
**Delivery of ceftriaxone within 1 hour**	16/239 (7%)	119/290 (41%)	<0.001
**Oxygen saturation >94% by 6 hours**	185/207 (89%)	253/268 (94%)	0.057
Resolution of clinical features of shock* by 6 hours	89/165 (46%)	99/182 (54%)	0.92
**Control of acute seizures by six hours**	29/31 (93.5%)	51/54 (94%)	1.0
**Transfusion of severe anaemia in emergency department**	0/8 (0%)	8/19 (42%)	0.04
**Blood glucose >4.0 mmol/L by 6 hours**	Not done	5/11 (40%)	NA

* Clinical shock defined as: heart rate of >100 beats per minute, systolic hypotension of <90mmHg or mean arterial blood pressure of <70mmHg, capillary refill time of >3 seconds, blood lactate of >4mmol/L or tachypnoea >25 breaths/minute

### Attainment of clinical goals in proven or probable bacterial meningitis

As in the entire study cohort, composite target analysis showed that GDT substantially achieved higher proportions of clinical goals identified in patients in this group compared to routine care (S5 and S6 Tables).

### Clinical outcome of patients with suspected, proven or probable ABM

**Fig 2:**
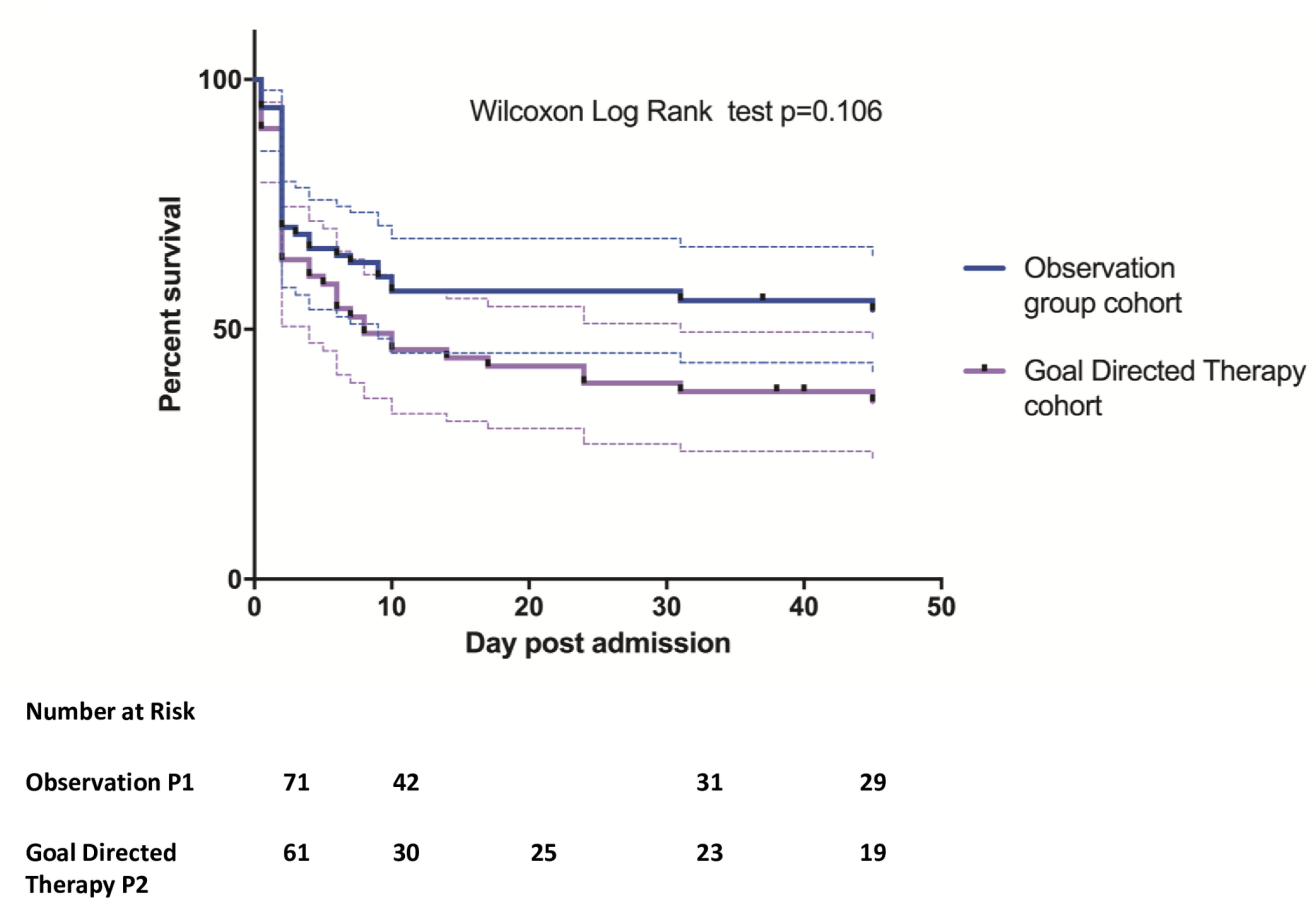
Survival curve of patients with proven or probable bacterial meningitis.

Case fatality rates (CFR) at 48 hours in all patients with suspected ABM was 31/273 (11%) in P1 and 43/290 (15%) in P2 (p = 0.31). CFR at 48 hours in proven/ probable ABM were substantially higher, 20/71 (28%) in P1 and 22/61 (36%) P2 (p = 0.33). Fourteen patients in P1 and one patient in P2 were lost to follow up between discharge and day 40. At day 40, CFR in proven or probable ABM with known outcome was 28/57 (49%) in P1 and 38/60 (63%) in P2 (p = 0.13). Right-censored survival analysis did not reveal a difference between the two phases (Fig 2).

Using a composite end-point of death or disability (mRS ≥2), GDT was associated with significantly higher rates of poor outcome at Day 10 (33/71 (46%) in P1 vs. 44/61 (72%) in P2; p = 0.004) (S7 Table), no differences were observed at day 40 (29/57 (51%) in P1 vs. 38/60 (63%) in P2; p = 0.19) (S7 Table).

## Discussion

We show that GDT for suspected ABM in an emergency department in sub-Saharan Africa is feasible and is associated with improved delivery of protocolised care. In line with recent studies of sepsis care bundles from Uganda, Zambia and Tanzania; adherence to the care bundle was good and patients received more interventions, particularly antibiotics and parenteral fluids [22, 23] [40]. Although a secondary outcome in a non-randomised study, the equipoise between routine clinical care and GDT in our study was not anticipated.

Patient characteristics in both phases of the study were similar, MAMS scores were not different between the groups in proven/probable meningitis [2, 36]. Early antibiotic therapy, airway support, seizure control and basic resuscitation for clinical shock are considered best practice in bacterial meningitis [21, 25], evidence exists from observational studies associating early antibiotic therapy with improved outcome [41]. Indeed, using data from earlier studies of ABM in Malawi, we have shown associations between clinical delay, abnormal physiology and a poor outcome, which are potentially amenable to GDT [2].

Care bundles are widely used for the management of sepsis in resource-rich settings, where reports of improvements in outcome have been variable. The Rivers 2001 trial demonstrated a substantial improvement in mortality from sepsis using an aggressive protocol with perfusion targets based on central venous lactate [18]. Recently, three large multi-centre randomised trials (PROCESS, PROMISE and ARISE) reported no difference in sepsis outcomes between Rivers protocol GDT, modified GDT, and routine care [42–44]. All three reported a significantly lower mortality rates (CFR 18–20%) across all groups including routine care at follow up, compared to Rivers (CFR 46% with routine care and 30% with GDT) [42–44]. Meta-analyses of randomised GDT trials in sepsis have been limited by significant heterogeneity across study designs, but show no clear outcome benefit for GDT [45, 46]. Most recently, a detailed analysis of 19 998 patients enrolled in over 4000 studies of GDT suggested that GDT may be associated with increased mortality in patients with high-disease severity [47]. Reasons for the discrepancies in mortality between the earlier and later studies are unclear but widespread incorporation of sepsis guidelines into the standardised care may have improved overall sepsis outcomes through emphasising early administration of antibiotics [47, 48].

The lack of efficacy of GDT and adjunctive therapies such as dexamethasone and glycerol [14] [15] in ABM in Malawi and other similar settings may in part be due to patients presenting late in the evolution of their disease and therefore no longer amenable to intervention. However, ABM patients present with a range of disease severities suggesting that outcome improvements remain possible. Our recent analyses of the glycerol trial data suggest that the reported adverse effects of glycerol were particularly marked in ABM patients predicted to have a good outcome [36]. It is possible that GDT benefitted only some patient sub-groups or indeed was harmful in those with severe disease [47]. Patients in the GDT group received more clinical interventions including parenteral fluids, due to the bundled nature of the intervention we cannot demonstrate if an individual component was harmful. All GDT sepsis studies from SSA have reported mixed outcomes, a study in Zambia was stopped early due to increased mortality in a sub-set of patients with hypoxaemic respiratory failure [22]. GDT was reported to improve outcomes from sepsis in Uganda in a non-randomised study, but no overall effect was reported in a mixed population of ICU patients in Tanzania [23, 40].

Limitations of our study include notable absences from our bundle including high flow oxygen and intensive care support [26]. To test GDT delivery, we had a higher nurse: patient ratio than is routine. We did not randomise patients following recommendations from the Medical Research Council (UK), randomisation is associated with risks of selection, allocation and concealment biases in single site studies [29, 49]. The study was carefully designed to minimise biases, patients were well matched. The observational design and loss of patients to follow up in this resource-limited setting limits definitive conclusions regarding the lack of efficacy of GDT on outcome [50].

The safety of translating GDT protocols from resource-rich settings where patients have access to considerably more human and therapeutic resources to settings in sub-Saharan Africa must be re-assessed. We suggest that while the principle of protocolised clinical intervention in SSA may be correct, lack of on-going monitoring, access to mechanical ventilation and critical care nursing may mitigate any benefit of GDT for both sepsis and ABM [22, 23, 34, 51, 52]. Data from large, randomised studies are required.

## Conclusion

Nurse-led GDT for suspected ABM is feasible in a resource-limited environment in sub-Saharan Africa. The use of interventions, tested in resource-rich settings, in SSA must be very carefully considered, given the substantial differences in populations, associated complications, worse outcomes and lack of access to on-going intensive care support. Better understanding of the pathophysiology and causes of mortality from meningitis in adults and adolescents in SSA is required to support the further design and testing of resource-appropriate ABM-specific interventions.

## Supporting information

S1 File Study protocol. (DOCX)

S2 File TREND checklist for trial reporting. (PDF)

S1 Table Physiological and laboratory characteristics on admission of all screened patients with suspected bacterial meningitis. (DOCX)

S2 Table Final diagnoses of all screened patients with suspected bacterial meningitis. (DOCX)

S3 Table Summary of CSF and blood culture results for all screened participants with suspected bacterial meningitis. (DOCX)

S4 Table Baseline characteristics of study participants with proven or probable bacterial meningitis. (DOCX)

S5 Table Composite achievement of clinical targets between Phase 1 and 2 for proven or probable bacterial meningitis. (DOCX)

S6 Table Clinical targets achieved by phase for patients with proven or probable bacterial meningitis at the end of 6 hours. (DOCX)

S7 Table Outcome of patients with suspected bacterial meningitis; and patients with proven or probable bacterial meningitis. (DOCX)
