# Supplementary material for: Goal directed therapy for suspected acute bacterial meningitis in adults and adolescents in sub-Saharan Africa
Source: PLoS One. 2017 Oct 27;12(10):e0186687. doi: 10.1371/journal.pone.0186687 (PMC5659601; doi:10.1371/journal.pone.0186687)
Supplement: S1 File — (DOCX) [file pone.0186687.s001.docx]

**Early goal directed therapy for adult bacterial meningitis in Malawi**

**Research Protocol**

**Research study leading to the submission of a PhD thesis**

PhD student Dr Emma Wall MBChB MRCP DTM&H MRes

Supervisor1. Professor RS Heyderman

Supervisor 2. Professor DG Lalloo

**Version 7**

Submission date: 1^st^ November 2010 COMREC study approval date January 2011

**Table of contents**

| **Section** | **Content** | **Page** |
| --- | --- | --- |
| Introduction & summaries |  | 3 |
|  | Details of investigators and collaborators | 3 |
|  | Executive Summary | 4 |
|  | Background and introduction | 6 |
|  | Rationale for study | 7 |
| Methodology |  | 8 |
|  | Objectives | 8 |
|  | 1. Study design | 8 |
|  | 1. Study place | 9 |
|  | 1. Study population (inclusion and exclusion criteria) | 9 |
|  | 1. i) Intervention design | 11 |
|  | ii) Endpoints | 13 |
|  | iii)Proposed laboratory tests | 13 |
|  | iv) Methodology for Real-Time PCR | 15 |
|  | v) Rationale and process for stored samples | 15 |
|  | vi) Recruitment and follow up | 16 |
|  | vii) Consent | 16 |
|  | viii) Study monitoring and adverse events | 17 |
|  | 1. Study duration and timing | 18 |
|  | 1. Sample size justification | 19 |
|  | 1. Data collection and management | 19 |
|  | 1. Data analysis including statistics to be used | 20 |
|  | 1. Results presentation | 21 |
|  | 1. Results dissemination | 21 |
| Ethical Considerations |  | 21 |
|  | Consent | 21 |
|  | Confidentiality | 22 |
|  | Burdens on study participants and host institution | 23 |
|  | Before/after study design | 24 |
|  | Sustainability | 24 |
|  | Storage, disposal and further use of clinical samples | 24 |
|  | Possible constraints | 25 |
| Requirements & budgetary support |  | 25 |
|  | Requirements | 25 |
|  | Budgetary estimates | 27 |
|  | Justification of the budget | 28 |
|  | Summary | 28 |
|  | References | 28 |
|  | Appendices | 31 |

**Investigators**

**Principle Investigator**

Dr Emma Wall MBChB MRCP DTM&H MRes (postgraduate student)

Malawi-Liverpool-Wellcome Clinical Research Programme

Po Box 30096 Chichiri

Blantyre, Malawi

[emma.wall@liv.ac.uk](mailto:emma.wall@liv.ac.uk)

+265 876 444

**Investigators and PhD supervisors**

| **Malawi** | **United Kingdom** |
| --- | --- |
| Professor Rob Heyderman  FRCP, DTM&H PhD  Professor of Tropical Medicine  & Director  Malawi-Liverpool-Wellcome Research Unit  Po Box 30096 Chichiri  Blantyre  Malawi  [rheyderman@mlw.medcol.mw](mailto:rheyderman@mlw.medcol.mw)  +265 +265 1876444 | Professor David Lalloo  FRCP DTM&H PhD  Professor of Tropical Medicine & Director Liverpool Wellcome Trust Clinical PhD programme  Liverpool School of Tropical Medicine  Pembroke Place  Liverpool, L3 5QA, UK  [dlalloo@liv.ac.uk](mailto:dlalloo@liv.ac.uk)  +44 151 705 3179 |

**Collaborators**

| Dr Theresa Allain, PhD FRCP  Head of Department of Medicine  College of Medicine, Blantyre  Malawi | Brigitte Denis  Head of MLW Microbiology  Malawi-Liverpool-Wellcome Research Unit  Malawi |
| --- | --- |
| Dr Dean Everett BSc PhD  Reader in Molecular Microbiology  Malawi-Liverpool-Wellcome Research Unit  Malawi | Dr John Chipolombwe  Research Registrar  College of Medicine, Blantyre  Malawi |
| Dr Mulinda Nyirenda  AETC Head of Department, Ministry of Health, Malawi |  |
| Dr Stephen Gordon FRCP PhD DTM&H  Reader in Respiratory Immunology  Liverpool School of Tropical Medicine, UK | Professor Tom Solomon PhD FRCP DTM&H  Professor of Neuroscience  University of Liverpool, UK |
| Professor Mike Levin FMed Sci FRCP&Ch PhD  Professor of Paediatric Infectious Diseases  Imperial College, London UK | Dr Brian Faragher  Senior lecturer in medical statistics  Liverpool School of Tropical medicine, UK |

**Institutions under whose auspices the research will be conducted**

1. Malawi-Liverpool-Wellcome Trust laboratories, Chichiri, Blantyre, Malawi
2. The College of Medicine, University of Malawi
3. Queen Elizabeth Hospital, Chichiri, Blantyre, Malawi

**Executive summary**

**Title**

Early goal directed therapy for adult bacterial meningitis in Malawi

**Type of research study**

Prospective clinical trial of early goal directed therapy using a before/after design

**Background**

The mortality rate associated with adult bacterial meningitis (ABM) in Malawi is 54% regardless of HIV status and has not been impacted by two adjunctive interventions tested in randomised controlled trials at Queen Elizabeth Central Hospital (dexamethasone and glycerol). A recent study has shown that delays within the medical care in QECH may contribute to this adverse outcome, such as delay to receive antibiotics. By starting a programme of triage, rapid resuscitation and treatment of sick children (ETAT) the overall inpatient mortality rate in acute paediatric admissions at QECH has been reduced from 18% to 9%. This approach, known as resuscitation delivered through early goal directed therapy (EGDT) has shown a relative mortality reduction from adult sepsis in developed countries by 25% (Absolute Reduction 9%), but this has not been tested in adults in Africa. This study seeks to investigate if by utilising existing resources to optimise delivery of standard clinical care interventions using a EGDT, the outcome from ABM can be improved.

**Research question** Can early goal directed resuscitation provided through a bundle of standard interventions have an impact on the clinical management and outcome in acute adult bacterial meningitis (ABM) in Malawi?

**Objectives**

**Broad main objective**

Assessment of the efficacy of early goal directed therapy (EGDT) to deliver target directed resuscitation using a ‘care bundle’ in a resource limited setting for adults with suspected bacterial meningitis

**Specific objectives**

1. To assess the efficacy of each clinical target in the clinical bundle
2. To assess the relative reduction in mortality due to ABM in adults receiving the bundle compared to controls
3. To estimate if the bundle has an impact on the incidence of acute seizures and neurological disability in survivors of ABM
4. To quantify the bacterial load of all patients with *S. pneumoniae* meningitis in blood and CSF, comparing survivors and non-survivors as a marker of poor outcome.
5. To assess inflammatory and immunological biomarkers of severity and outcome in serum and CSF samples to better understand the pathogenesis of adult bacterial meningitis in this context

**Methodology**

1. We will test the bundle in a before/after study with sequentially phased prospective control and intervention groups in the new Anadkat-Wellcome Trust Adult emergency and Trauma Centre (AETC) at Queen Elizabeth Hospital. The bundle will include nurse led rapid assessment and triage and targeted delivery of resuscitation to all subjects with suspected ABM using clear, achievable goal directed targets. Subjects will be excluded retrospectively if an alternative diagnosis is made.
2. The bundle will only include well recognised standard treatments that are already available at QECH, but the targets will ensure these are given promptly and appropriately.

**Expected findings**

We expect that the clinical care of adults with suspected bacterial meningitis will be enhanced using the treatment delivered with EGDT, and the targets will be achievable. We expect this will translate to an improvement in mortality and reduce morbidity in survivors.

**Background information and introduction**

**Burden of adult meningitis**

Adult bacterial meningitis (ABM) is a life threatening condition, particularly in sub-Saharan Africa where the mortality is 50-70%(1-3).Two major trials conducted in Blantyre of adjunctive therapies (dexamethasone and glycerol) failed to show any mortality benefit, Ajdukiewicz 2009 (1) (un-published). Many of these patients present *in extremis*, and systematic delays within the hospital, including delays to see a doctor and receive intravenous antibiotics may also contribute to high mortality rates (Wall, Cartwright et al 2010 unpublished, data presented at the COM Research Dissemination Conference).

**Early goal directed therapy to improve clinical management**

Since a landmark study showing a benefit from targeted resuscitation in sepsis, the concept of using early goal directed therapy (EGDT) with simultaneous interventions (‘sepsis bundle’) has been shown to be highly successful in reducing mortality from bacterial sepsis and septic shock (4). EGDT consists of delivering existing, recognised, evidenced based treatments for a particular condition, in a rapid, protocolised fashion to achieve clinical targets, with the aim to optimise the clinical care available and therefore the outcome.

Estimates of the relative reduction in mortality with EGDT for adult septic shock are between 20-25% (absolute risk reduction 9-10%). The single most effective component has been shown to be early antibiotics (5). Goal directed fluid resuscitation and tissue oxygenation are also critical, forming internationally accepted standards for septic shock management worldwide (6). To be included in a bundle, all EGDT elements must have individual supportive evidence of efficacy and it is expected that the overall bundle, when delivered together, efficacy will be greater than the individual components (7).

**Early goal directed therapy in resource limited settings**

Using EGDT to improve survival is highly applicable to resource limited hospitals. In 2001, the Department of Paediatrics in Queen Elizabeth Central Hospital (QECH) developed Emergency Triage and Treatment (ETAT) in their clinic to prioritise the care of sick children, and have shown an overall reduction in mortality amongst emergency admissions from 18% to 9% (8). Rapid recognition and management of acute illness in adults is part of the 2004 WHO guidelines on the management of unwell adults in resource limited settings ‘Integrated management of adolescent and adult illnesses’ (9). Although neither EGDT nor ETAT are specifically mentioned in this publication, the principles that underline both of these methodologies are recommended. No current publications or registered clinical trials have tested the concept of EGDT in adults in Africa.

**Bacterial load as a marker for mortality in adult meningitis**

High bacterial loads in patients with *S. pneumoniae* sepsis and pneumonia (adults) and meningitis (children) have been correlated with poor outcome, but this has not been studied in ABM (10-11). Bacterial load may prove to be an important contributor to poor outcome in Malawi, and impacted on by early antibiotics. This data may also be important in the further planned laboratory research work, as correlating bacterial load with the degree of immunological response in the CSF may provide novel insights into the pathogenesis of ABM.

This study will test if the concept of EGDT for adults is possible in a resource limited setting, and if it has an impact on outcome. Based on the surviving sepsis campaign bundle (6) and an analysis of the clinical data from MLW/ QEH, this study will test a care bundle, designed to optimise the management of adult meningitis in Malawi, using existing resources in a clinical before/after study.

**Rationale/Justification for the research project**

**Current problems in delivering care for adult meningitis in Blantyre**

The mortality rate for adult bacterial meningitis is very high in resource limited settings, and to date there is no clear explanation for this, or for the failure of adjunctive treatments which have been shown to be effective in other settings. It appears likely from our preliminary studies at QECH that delays in delivering essential care such as antibiotics and intravenous fluids are major contributors to the poor outcome. It is also likely that delays in reaching hospital care also contribute, although this has not been proven.

**Applying EGDT to resource limited settings using existing treatments using targets is potentially achievable and has not been done in adult medicine in Africa**

It is very important to know if deficiencies in the existing healthcare structure contribute to poor outcome and if outcome can be improved by optimising care. By using the early goal directed target (EGDT) approach, we can test if through optimising existing resources and improving clinical care (by shortening assessment times and giving appropriate recognised treatments within a prompt timeframe) is achievable, and if any impact on outcome through this approach is possible.

**Summary of the clinical evidence to support each element of the care bundle**

All elements of the bundle have strong evidence to support their use in ABM, and are based on the surviving sepsis guidelines (6). All have been adapted to be feasible in a low income setting. Airway management has been shown to reduce the risk of airway obstruction in coma (12-13), and is essential for a patient with seizures. Naso-pharyngeal airways are safest where invasive ventilation is not available. Cerebral oxygenation and perfusion are essential in ABM. A 30°C head tilt has been shown to optimise perfusion and minimise oedema in brain injury (14), and oxygen saturations of less than 93% were shown to be associated with an increased risk of death in an analysis of mortality from meningitis in Malawi (Wall, Cartwright et al 2010 unpublished). Early antibiotics in meningitis have been shown to have a significant association with survival in several studies (15-17). Fluid resuscitation in ABM is more controversial, as no RCTs have been performed to investigate this. Fluid restriction in children is associated with harm in meningitis, and initial aggressive resuscitation in adult sepsis is associated with survival (6, 18). Therefore if adults appear clinically shocked, they will be resuscitated with fluid, but if not they will receive maintenance IV fluids for the duration of the intervention. Oxygen delivery to tissues in critically ill patients has been shown to decrease significantly when the haemoglobin is <7g/dL, and transfusion to a haematocrit of >30% forms a component of the surviving sepsis guidelines (6, 19). The WHO guidelines on blood transfusion state that a transfusion should be given when tissue hypoxia is compromised by anaemia (20). In an unpublished analysis of patients with ABM a haemoglobin of <6.0g/dL was significantly associated with increased mortality, and therefore this has been chosen as an appropriate transfusion limit (Wall, Cartwright et al 2010).

**Use of the study results**

If the study is successful and the EGDT targets are achievable within the QECH setting, funding and ethical approval will be sought for a much larger cluster randomised study throughout Southern Africa to test the bundle concept definitively.

**Objectives**

**Broad main objective**

Assessment of the efficacy of early goal directed therapy to deliver target directed resuscitation in a resource limited setting for adults with suspected bacterial meningitis using a clinical care bundle.

**Specific objectives**

1. To assess the efficacy of each clinical target in the clinical bundle
2. To assess the relative reduction in mortality due to ABM in adults receiving the bundle compared to controls
3. To estimate if the bundle has an impact on the incidence of acute seizures and neurological disability in survivors of ABM
4. To quantify the bacterial load of all patients with *S. pneumoniae* meningitis in blood and CSF, comparing survivors and non-survivors as a marker of poor outcome.
5. To assess inflammatory and immunological biomarkers of severity and outcome in serum and CSF samples to better understand the pathogenesis of adult bacterial meningitis in this context

**Methodology**

1. **Study design**

The study is a single site, clinical prospective observational study with a before/after design, which is the only feasible study design with the current constraints of ethics, time and funding. A traditional randomised controlled design is not appropriate for this intervention. Each element of the clinical care bundle has evidence to support its use and forms part of standard care. Therefore it is unethical to randomise half of the study population not to receive good quality medical care. However a prospective control population is needed as the potential additional benefit to participants of admission through the AETC is not yet quantified, compared to admission directly through the medical ward. The only alternative to a parallel design for a complex intervention is a before/after where the benefit of the intervention can be assessed in the after group, compared to the control before group. There are several problems with this design, mainly due to several confounders including time and staff. The ideal design for this study is a cluster randomised study, where the intervention would be tested in several centres, and outcomes compared to control centres without the intervention. This is the only study design that can give definitive statistical evidence of efficacy of EDGT. However this type of study is large and time consuming and beyond the scope of this fellowship. Should the EGDT intervention prove successful in this study it will act as a pilot for a cluster randomised study to be performed throughout Southern Africa.

The before/after design is the most common for studies testing EGDT in sepsis, as it is the only methodology that can be used in a single site. In 9 studies of EGDT summarised in a recent systematic review, 8 were single site before/after and one was a cluster randomised design over multiple sites (4-5).

The Medical Research Council guidelines on trial design for complex interventions, endorse the before/after design where a group of interventions are given together in a single site and every attempt is made to minimise any aspects of study delivery and design that may lead to confounding (21).

This before/after study is divided into two consecutive phases, the ‘before’ group named phase 1(observational controls) and the ‘after’ group, phase 2 (implementation of bundle). Phase 2b will start at the same time point as phase 1, but one year later to minimise the effect of seasonal variation in the presentation of ABM.

All adults admitted to AETC in both phases with suspected ABM will be screened and included using identical inclusion criteria to ensure the participants in both phases are as similar as possible.

To minimise confounding, active recruitment, prospective data collection and follow up of all study subjects will be identical in both phases.

Phase 2 is divided into 2a, a short pilot phase to introduce each bundle element sequentially and phase 2b, the active bundle. This is due to the complexity of the care bundle to ensure that all study team members are familiar with each target and intervention before full active data collection starts in Phase 2b. Multiple studies of care bundles in different settings have documented the failure of implementation of a bundle if it is not piloted first and all study staff are trained fully in the bundle delivery (22-23).

1. **Study place**

This study will be based entirely in the new Anadkat-Wellcome Trust Adult Emergency Trauma Centre (AETC) which is was completed in September 2011 and became operational in October2011 and Queen Elizabeth Central Hospital, Blantyre, Malawi

1. **Study population**

Adults (aged 14 or over) presenting to the AETC with suspected bacterial meningitis

**Inclusion criteria**

- Adults ≥14 years age
- Fever/history of fever plus one of:

1. Coma
2. Severe Headache
3. Nuchal rigidity
4. Seizures
5. Confusion

The diagnosis of ABM will be based at presentation on clinical symptoms and signs. A formal diagnosis of ABM will be made following CSF analysis using the following criteria: ***CSF WCC >50 cells/mm^2^ (predominantly neutrophils), visible bacterial seen in the counting chamber irrespective of cell count***, ***positive Gram’s stain*** or culture/PCR positivity for any bacteria known to cause meningitis.

**Exclusion criteria**

Immediate

- Pre-admission diagnosed terminal illness (e.g. metastatic malignancy)

LP results

- CSF positive on microscopy, culture or antibody testing for an organism known to cause chronic meningitis (fungal/tuberculosis/parasitic etc.)
- CSF that is culture/microscopy/PCR negative, but the CSF WCC is greater than 80% lymphocytes and no pre-admission antibiotics have been given

Subjects with non-inflammatory ***CSF < 50 WBCs*** and culture/gram/PCR negative will be excluded from the main ABM analysis, but will be analysed as a separate group.

Patients presenting in-extremis or with symptoms or signs suggesting death within 4-6 hours will be included in the analysis, but also subjected to a separate sub analysis.

**Control group**

Phase 1 will consist entirely of recruitment of controls for the ‘before’ phase 1 of the study design. All aspects of recruitment and the consent process will be identical to the intervention ‘after’ phase 2. No EGDT will be given to the controls, and their clinical management will be determined by the treating clinician and delivered by the study nurses. Regular observations will be performed and charted by the study nurses for each subject for the 6 hour study period.

**Intervention group**

Phase 2 will consist entirely of recruitment of subjects to be given the care bundle intervention. All other aspects of recruitment consent and observation will be identical to phase 1.

1. **Detailed Methodology**

**iv. i Intervention design**

EGDT will be delivered using a clinical care bundle, based on the ‘ABCD’ approach to resuscitation which has been developed and validated by the American and UK resuscitation councils (24) and consists of eight components, all of which are currently available in QECH and all of which are recognised existing clinical treatments which have supportive evidence of efficacy. The individual components of the bundle can be seen in table 1.

Staff training will form an important component of bundle delivery during phase 2a and ongoing through phase 2b, but will not form a specific bundle element. This is due to difficulties in assessing staff training when no formalised resuscitation training is available that is appropriate to resource limited settings.

The duration of the intervention will be 6 hours from initial screening to the point at which attempts to meet all targets have been achieved. Each subject will receive hourly observations including GCS scoring, blood pressure and pulse measurements. A lumbar puncture for the diagnosis of ABM will be performed at the earliest opportunity.

Study nurses and a clinical officer will deliver the bundle following training, using defined targets (table 2) within the AETC. At the end of the 6 hour study period, the bundle will stop, and the subject will be transferred to the medical ward for ongoing routine care. A flowchart of the study process is available in the appendix 9.

***Table 1 – Bundle components***

| **Bundle components** |
| --- |
| Naso-pharyngeal airway if GCS <8  Head up/bed tilt 30° if GCS <11  IV access, 2g Ceftriaxone stat  Oxygen via concentrator if SpO2 <93%  IV fluid (Ringer’s Lactate) bolus 20ml/kg if clinical shock, 125ml/hr if no clinical shock (table 3)  Transfusion of packed red cells if haemoglobin <6.0g/dL  Correction of hypoglycaemia with oral dextrose and drinks/food if GCS >11, or with iv dextrose 10-50% via a bolus/infusion if GCS <11  Prompt treatment seizures  *(IV diazepam/lorazepam plus phenytoin for persistent fits/status epilepticus)* |

***Table 2 – Goal directed targets***

| **Parameter** | **Target** | **Intervention** | **Test of intervention** |
| --- | --- | --- | --- |
| Timing of clinical assessment | Medical review <1 hour of arrival | Training in recognition of the symptoms and signs of meningitis and rapid triage | Timed and signed flow chart |
| Antibiotic therapy | 1^st^ dose within 1 hour of arrival | Education of importance of antibiotics, sepsis flow chart | Timed drug chart |
| Glucose | BM >4 | S/L , NG or IV dextrose | Repeat BM |
| Oxygenation | Sp02 >94% | Nasal flow 02 from concentrator  Packed red cell Transfusion if significant anaemia (Hb 6.0g/dL) | Regular Sp02 monitoring |
| Perfusion | CRT<2 sec, BP >90 syst, MAP >70, no postural hypotension (lying-sitting)/  UOP>0.5ml/kg (if catheterised | IV fluid bolus 20ml/kg Ringer’s lactate where available. | Repeated fluid balance assessment at 1hour then repeated |
| Seizures | No seizures | Acute prompt treatment of seizures with IV/PR benzodiazepines and IV phenytoin if seizures persist | Seizure chart |

***Table 3- Definition of clinical shock***

| **General variables** | **Hemodynamic variables** | **Organ dysfunction variables** | **Tissue perfusion variables** |
| --- | --- | --- | --- |
| Heart rate >90 bpm | Arterial hypotension (SBP <90 mm Hg | Acute oliguria (urine output <0.5 mL/Kg/hr **or** <45 ml/hr for at least 2 hrs (if catheterised)/ no urine production for 12 hours despite adequate fluid resuscitation) | Decreased capillary refill (>3 seconds) |
| Tachypnea RR>25 | MAP <70 mm Hg | Ileus (absent bowel sounds) | Skin mottling |
| Altered mental status | SBP decrease >40 mm Hg |  |  |

All interventions are part of routine accepted care for critically ill patients at QECH, however they are incompletely implemented and are currently not target driven.

**iv.ii Endpoints**

***Primary***

Proportion of each clinical target achieved by the bundle

Total proportion clinical targets when combined together, achieved by the bundle

***Secondary (to be measured at day 10 and day 40)***

1. Death
2. Persistent seizures requiring treatment
3. Significant neurological disability
4. Functional ability
5. Evidence of persistent renal injury
6. Blood and CSF bacterial load of *S. pneumoniae*

Death will be measured as a composite secondary endpoint (days to death/last known alive) using the following methods:

*Day 10 (acute)* – date of discharge (alive) or date of death will be recorded up to 10 days of inpatient antibiotic therapy.

*Day 40 (convalescent)* - Final outcome recorded ‘alive or dead’ following records from follow up or response to mobile telephone at until 6 weeks.

A history of seizures and functional ability since discharge will be taken, and endpoints 3-5 will be measured formally using the ***Modified Rankin score***, serum creatinine (to calculate eGFR), blood pressure and urine dip (for protein).

**iv.iii Proposed laboratory tests**

We propose all subjects recruited in both phases will have the following tests. All subjects will also have CSF and serum stored for testing with Real-Time PCR to quantify the bacterial load at the end of the study.

| **Tissue type** | **Justification** | **Volume required** |
| --- | --- | --- |
| **Cerebrospinal fluid (CSF)** |  |  |
| Microbiology (microscopy, protein, glucose, bacterial/fugal/TB culture) | Clinical diagnostics(25) | Maximum volume  5-15mls |
| Real-Time PCR | Clinical diagnostics and potential marker of poor outcome(26) |  |
| Storage | See objective 5, future research into biomarkers in meningitis.  No additional sample will be taken for storage, only the remainder of samples taken for clinical diagnostics and surplus to clinical requirements will be stored |  |
| **Blood for rapid diagnostics (finger prick tests on admission)** |  |  |
| Lactate | Marker of septicaemia(27) | 1 drop |
| Glucose | Risk factor for disease severity and seizures(28) | 1 drop |
| Haemoglobin | Marker of severity and to determine transfusion requirements (Wall, Cartwright et al, unpublished) | 1 drop |
| **Blood (laboratory tests)** |  |  |
| Sodium (1ml) | Risk factor for seizures and disease severity(29), and important in clinical management. This can be performed on the same sample taken for creatinine and HIV antibodies, minimising the overall volume required (3ml maximum in 1 tube) | Maximum volume required 20mls |
| Creatinine (1ml) | To determine if acute renal failure is present (on admission and again at follow up)(30) |  |
| Full Blood Count (2ml) | Clinical diagnostics |  |
| HIV antibodies when consent given (1ml) | Clinical diagnostics |  |
| Malaria thick film (1ml) | Clinical diagnostics. This can be done on the same sample for a full blood count, using one tube with a maximum volume of 3ml. |  |
| Blood culture (10ml) | Clinical diagnostics. 10ml is the optimal volume to ensure circulating bacteria are detected. |  |
| Real-Time PCR (1ml) | Clinical diagnostics and potential marker of poor outcome. 100ul of serum is required for RT-PCR, so 1 ml of whole blood will yield this. |  |
| Storage (2-3ml) | See objective 5, future research into biomarkers in meningitis.  Exploration of the host genotypic and immunological response (No additional sample will be taken for storage, only the remainder of samples taken for clinical diagnostics and surplus to clinical requirements will be stored) |  |
| **Urine** | Dip for proteinuria (5mls) |  |
| **Nasal swab** | Culture for *S. pneumoniae* and genotypic exploration of micro-evolution between the nose/blood and CSF of *S. pneumoniae* | 1 swab |

**iv.iv Methodology to address specific objective 4: Quantification of the *S. pneumoniae* bacterial load**

Serum and remainder CSF (following diagnostic procedures) will be removed from each subject with informed consent and stored at -80°C in the MLW laboratory. The diagnostic CSF algorithm will be followed for the study (appendix1). All samples that are gram stain/culture positive for *S. pneumoniae* or inflammatory but culture negative will undergo DNA extraction and Real-Time PCR using pneumolysin primers with TAMRA fluorescent probes. Standards will be generated using synthetic pneumococcal DNA (Eurofins, USA).

These results will be added to the trial database and analysed with outcome comparing survivors and non survivors between phases 1 and 2.

**iv.v Methodology to address objective 5: Further research use of remaining samples**

Specific permission will be requested from each participant during the formal informed consent process for the further use of any remaining tissue samples. We plan to analyse these samples in a future series of experiments to explore the host immunological response to the pathogen *S. pneumoniae*, including measurement of markers of apoptosis and complement activation, based on work at LSTM (31). Dr Stephen Gordon’s laboratory at the LSTM is currently studying the host immunology of pneumococcal disease, with an aim to novel protein vaccine design. A recent analysis of the CSF and serum samples from a previous meningitis trial in QECH, the steroids in adult meningitis (SAM) trial (1) has shown that there is massive complement consumption within the CSF compartment in ABM (work currently un-published). This is previously un-described in meningitis and may indicate a novel aspect of pathogenesis. Samples from this study would be used in experiments to explore complement consumption further. We will also request permission from the participants for the use of genetic material (RNA) to be analysed (both from bacteria cultured and from the host), in a series of collaborative experiments studying gene expression in ABM with and Imperial College. A large proteomic analysis of CSF samples from the SAM trial failed to identify any biomarkers of disease severity (31). We plan to analyse gene expression in the serum and CSF, to identify if severity is due to gene over or under-expression, and to use this approach to identify disease severity biomarkers and guide further proteomic work. A material transfer agreement is attached for this work. Recent work from Professor Levin at Imperial College using gene expression has shown an important genetic basis for meningitis in Europe due to *Neisseria meningitidis*(32). No studies to date have studied this in meningitis due to *S. pneumoniae*, or used gene expression to identify patterns of disease susceptibility in resource limited settings. All additional research work under objective 5 using stored samples from this study is aimed at understanding the very high mortality from ABM in Africa, and with the long term aim of designing appropriate vaccines and adjunctive therapies to improve outcome within Africa. All samples will be stored for a maximum of 5 years. Where laboratory technology permits, the majority of this work is planned to be done in Malawi, providing capacity building, training and educational support to local staff and adding to the research profile of Malawi.

**Summary of sample storage and disposal.**

| **Sample** | **Storage** | **Duration** | **Disposal** |
| --- | --- | --- | --- |
| Blood | MLW -80oC freezer and  LSTM -80oC freezer if samples moved under a MTA. Stored for future related projects with full consent of study participants | Stored up to 5 years after the end of the project. | Usual Category II waste procedure according to University of Liverpool/Liverpool School of Tropical Medicine protocol |
| **Sample** | **Storage** | **Duration** | **Disposal** |
| CSF | MLW -80oC freezer and LSTM -80oC freezer if samples moved under a MTA for further related collaborative research studies with full consent of study participants. | Stored up to 5 years after the end of the project. | Usual Category II waste procedure according to University of Liverpool protocol/ Liverpool School of Tropical Medicine protocol |

**iv.vi Recruitment and follow up**

**Recruitment**

Subject recruitment in both phases will be entirely within the Adult Emergency and Trauma Centre (AETC) at Queen Elizabeth Central Hospital (QECH). Subjects will be recruited between 8am and 4pm by a team of 6 study nurses based in AETC. The nurses will screen all unwell triaged patients by the inclusion criteria and recruit, following verbal assent, all adults with suspected ABM.

Based on 9 years (2001-9) of data from MLW, it is expected that 50-100 cases of proven ABM will be recruited per 6 months, and a total of 1-2000 potential trial participants will be screened. Flexibility is built into the study timeline to allow for slower/delayed recruitment, particularly during the introduction of the bundle in phase 2.

**Follow up**

All participants who have been included based on CSF results will be followed up on the medical ward during their inpatient stay. Each participant will be reviewed daily and clinical progress data will be collected by the PI, the study clinical officer or one of the study nurses. The results of blood tests performed by the inpatient medical team will also be collected by download from the Spine recording system. For more detail on Spine please see data confidentiality on page 23.

All included subjects from Phases 1 and 2 surviving to discharge from hospital will be followed up weekly for 6 weeks (40 days) by text message sent to a nominated mobile phone (with consent from the participant). At six weeks each surviving participant will be invited to a study outpatient appointment at QECH. The outpatient follow up clinic will be staffed by the PI and the study clinical officer. Clinical data will be collected in outpatients to inform the secondary endpoints of the study.

**iv.vii. Consent**

The aim of the bundle is speed of onset, and delays inherent in the informed consent process have the potential to impact on the success of the treatment. For most published studies testing sepsis bundle implementation using a before/after design, the need for informed consent was waived at institutional ethical review (27, 33-37). One study identified did not obtain informed consent from participants, and did not discuss any ethical concerns, or disclose institutional ethical review (38). However, we do not consider this approach appropriate for this setting.

We propose to use a two stage, delayed formal informed consent to enable the rapid onset of the bundle delivery. The consent process will be GCP compliant, and will consist of two steps: verbal assent by the participant or their guardian on admission and recruitment, and full informed consent when the subject is stabilised. This is planned for two main reasons. Firstly, the process of verbal assent to gain basic agreement for participation will facilitate rapid intervention. This followed by formal informed consent for ongoing participation at a later date is a standardised practice, recommended by the FDA and has been used in many trials of emergency interventions. Secondly, potential study participants are likely to be critically unwell on arrival at the hospital, and may have impaired consciousness, and therefore will not fulfil the requirements for standard GCP informed consent. This has been a problem for all studies that investigate critical illness testing rapid delivery of the intervention to a severely unwell participant.

**Verbal assent**

As the subject is identified as a potential study subject at triage in AETC, the recruiting study nurse will give verbal details of the study, and request verbal consent for inclusion ‘verbal assent’. If the answer is affirmative, the subject will be recruited and monitoring/bundle will be started immediately. An assent form will be signed at this point by the study nurse and if possible the patient or carer.

**Delayed formal informed consent**

Once the subject is stabilised, and able to talk (or a carer is present), formal written informed consent will be requested. If no carer is present and the patient is comatose, the subject will be included on the basis of best interests, until a time when a carer can be located to request consent.

No samples of tissue will be analysed, excepting those necessary for acute clinical management, until written informed consent has been given. If consent is denied at this stage then all records of that subject will be deleted from the main trial database. If the subject dies before either verbal assent or written informed consent can be given then data collected to that point will be used in the study and the carers will only be asked for written consent for the use of tissue samples in research laboratory studies

**Requirements for GCP informed consent**

GCP formal informed consent can only be requested when the subject is physically and emotionally well enough to receive and comprehend the details given to the subject in the information sheet and by the study worker. Due to the high likelihood of comprehension being impaired during an acute episode of ABM, it is not appropriate to take informed consent at the acute admission point. Once the subject has been stabilised, and has improved to a point where s/he has reasonable comprehension and retention of information, formal written informed consent will be requested. However the bundle will need to be started before informed consent can be obtained.

**No new drug or treatment is being tested that requires informed consent to administer**

The bundle consists of normal, recognised treatments, each with evidence of efficacy that do not require informed consent to administer in standard clinical practice.

**iv.viii Study monitoring and adverse events**

**Data monitoring**

A formal data monitoring committee is not required for this study, as the interventions being tested have individually been proven to be effective, and there is no blinded/randomised intervention. However Professor Tim Peto (Professor of Tropical Medicine, University of Oxford) has agreed to act as an independent study monitor and review monthly aggregated reports on all outcomes and SAEs. A Malawian study monitor is due to be appointed.

**Risk of serious adverse events**

As no new intervention is being tested, the study team will be familiar with all elements of the bundle, and will receive additional training by the PI in both safe administration of each element and awareness of the potential for harm. Therefore the risks to the participants associated with the study will be minimal. Each participant will be very closely monitored with regular clinical observations throughout the 6 hour study period, and any clinical deterioration (based on the medical alert system) will trigger a medical review with the PI. The PI will be available in person or by telephone during all study hours to discuss any problems or issues and to review any deteriorating participant. For problems out of hours the medical on call team will be called to review the participant. When a clinical deterioration is noted and managed, the PI will review in detail all of the case records of that participant.

**Process of detecting and reporting SAEs**

To ensure adverse events are detected at an early stage, monthly mortality figures will be sent to the trial monitor for review. This data will be aggregated over time to improve interpretation and monitoring. Any suspected or unexpected serious adverse events (SAE/SUE) will be discussed immediately with the PI, study nurses and clinical officer and a record form will be completed. A serious adverse event will be classified as an unexpected negative outcome, harm to a trial participant, or an occurrence that results in a complaint to the trial team from a participant or their carer, or from within the AETC team.

All SAEs and SUEs will be discussed by the principle investigator with the chief investigators and details given to the trial monitor with aggregated mortality data. IRB COMREC will also be given details of all SAEs. If deemed necessary by either the CI, IRB COMREC or the study monitor, recruitment will be paused, and a meeting of the trial steering committee (TSC) convened to discuss the SAE and any protocol changes that may need to be made.

1. **Study duration and timing**

The study is planned to recruit for 2 years in total and 3 years in total is allocated for all formal stages of training, planning, recruitment, analysis and dissemination of the results. The detail of the relevant allocated times is represented in the following Gantt chart.

**Timetable**

1. November 2010-January 2011 Study set up in Malawi
2. Feb 2011-October 2011 Maternity leave
3. November 2011 Review of study set up so far.
4. Completion of data collection tools, SOPs and study logistics. Final equipment procured.
5. December 2011 Study staff start induction in the AETC, working as supra-numerary staff. Piloting of data collection tools, testing of all equipment and SOPS.
6. January 2012: Phase 1 prospective control recruitment starts
7. September 2012: Phase 1 recruitment ends.
8. September-December 2012: Phase 2a introduce and pilot the meningitis care bundle
9. January 2013: Phase 2b ( care bundle) recruitment starts to
10. September 2013: TSC meets to discuss Phase 2b results. Time allowed for continuation of 3-6 months.
11. December 2013: Study ends, results analysed. Further laboratory work completed.
12. May 2014 PhD write up, scientific papers and results analysis completed.

**Gantt chart of timetable/milestone reporting**

| **Study event** | **3 months** | **1 month** | **1 month** | | **9 months** | | **3 months** | | **9 months** | | | | **3 months** | | | | **9 months** | |
| --- | --- | --- | --- | --- | --- | --- | --- | --- | --- | --- | --- | --- | --- | --- | --- | --- | --- | --- |
| **Study setup** |  |  |  | |  | |  | |  | | | |  | | | |  | |
| **Final study organisation** |  |  |  | |  | |  | |  | | | |  | | | |  | |
| **Staff induction and pilot data collection** |  |  |  | |  | |  | |  | | | |  | | | |  | |
| **Phase 1** |  |  |  | |  | |  | |  | | | |  | | | |  | |
| **Phase 2a** |  |  |  | |  | |  | |  | | | |  | | | |  | |
| **Phase 2b** |  |  |  | |  | |  | |  | | | |  | |  | | |  |
| **Data analysis** |  |  |  | |  | |  | |  | | | |  | |  | | |  |
| **TSC meeting** |  |  |  | |  |  |  | |  |  |  |  |  |  | | |  | |
| **Milestone report** |  |  |  |  |  | |  |  |  | | | |  |  | |  |  | |

1. **Sample size**

In published data from resource rich hospitals, all EGDT studies have reported sample sizes from 100-500 in both cluster randomised and before/after designs. Two studies have demonstrated a mortality benefit with 100 subjects in each group using a before/after design (33-34). However it is unclear how this effect size can be translated to a resource limited African hospital, as no studies of EGDT in adults have been conducted in this setting and the ETAT paediatric data was retrospective with no sample size calculation.

We propose to recruit equal numbers of subjects in phases 1 and 2, and treat this study as pilot testing of EGDT. Using the projected timeframe I expect to recruit 200-250 subjects in total, equally distributed over phases 1 and 2. There is a risk with before/after studies that the design may over-estimate the actual size of any potential benefit from the intervention (39), and can be confounded by time. Therefore definitive conclusions as to the efficacy of the bundle can only be drawn from larger, cluster randomised parallel studies, and this study will provide preliminary proof of principle.

1. **Data collection and management**

**Clinical data**

Coded clinical data will be captured by the study nurses and clinical officer electronically into a secure electronic database using the ‘redcap’ system, and from there it will be transferred to statistical software. Patient identifiers will be used from the ‘Spine’ data recording system using barcodes, enabling data to be collected across multiple sites within QEH. See data confidentiality section for more detail on Spine page 23

Data will be imputed from the bedside into either a secure study laptop based in the AETC, or via a handheld computer (PDA) using a barcode reader, resulting in rapid and secure data collection. All data will be downloaded to the MLW secure server every 24 hours and will not be retained on the electronic devises to minimise the risk to confidentiality. No identifying details except the Spine data code will be retained in the database.

**Demographic data**

Basic identifying demographic data will be will be kept in a written record in a secure locked cabinet, separately from the main study database, and will provide a link to the barcode data. This is necessary to be able to text message/telephone study participants on a weekly basis to ensure the maximum survival information is collected for the study endpoints. This data will only be available to the study team and will be destroyed at the end of the study. No clinical data will be noted in this record.

**Target and bundle sustainability related data**

Data will be collected on a weekly basis by the nurses through feedback to the PI to assess if the AETC staffing and equipment levels would be appropriate to deliver targeted care. An assessment will be made with the assistance of a local health economist of the additional cost of implementing each element of the bundle (if proven to be effective) into long term practice.

1. **Data analysis**

Each element of the bundle will be analysed separately to calculate the percentage of each clinical target was achieved.

Analysis will be primarily by intention to treat, and then per-protocol. Data will be collected using ‘redcap’ electronically and then transferred to SPSS/PASW statistical programme and GraphPad5™ software for analysis.

**The following statistical tests will be used to compare the intervention and control groups.**

- Composite analysis of each proportion of target achieved
- Simple mortality proportions
- Kaplan-Meir survival analyses to establish the pattern of the death rate
- Chi squared tests and Fisher’s exact test will be used to analyse the significance of differences between the two phases of fixed endpoints.
- Logistic regression will be used to give an estimate of the size of the change in outcomes.
- Where appropriate the students T test and the Mann-Whitney test of medians will be used to compare the outcome between the bacterial loads in blood and CSF.

**Sub-analyses will be undertaken of the following groups**

1. Patients presenting where death is predicted by the admitting clinician within the bundle implementation time period (6-8 hrs).
2. Subjects with normal CSF and an alternative diagnosis such as sepsis
3. Subjects with abnormal CSF but negative microbiological cultures and PCR.

4 weeks of statistician time is included in the budget. Data from this study will be aggregated with previously collected adult meningitis data from 3 previous studies in Blantyre to continue to study long term changes in meningitis mortality.

1. **Results Presentation**

The results will be presented in a variety of formats. Simple bar charts and pie charts will be used to compare the proportion of each target achieved with the EGDT. Kaplan-Meir survival graphs will be plotted for mortality analysis and the subjects from the two phases will be compared. The remaining outcome data will be presented in table format and displayed using bar charts. All results will be displayed in formats using GraphPad5™ software

1. **Results Dissemination**

**Local dissemination**

The progress of the study and the final results will be presented in a series of teaching seminars to the staff of the AETC and the adult medical ward. They will also be presented to the research and teaching staff at the College of Medicine Research In Progress meetings and Research Dissemination Day. Where possible results will also be presented through the local media to inform study participants of the results. It is planned that through the dissemination of the results, and based on the study results and experiences of the study team, guidelines for the management of ABM in resource limited settings will be written and the principle of EGDT will form part of the ongoing teaching to both student and qualified doctors and nurses in the College of Medicine (continuing medical education CME). Copies of the formal report from this study will be presented to COMREC, the College of Medicine Library, the Health Sciences Research Committee, and the University Research and Publication Committee.

**International dissemination**

A formal report will be presented to the funding body, The Wellcome Trust, and a copy given to COMREC. The results will be presented to the Liverpool School of Tropical Medicine in the form of a thesis and will be submitted for a doctoral degree by Emma Wall.

The formal results of the trial are also planned for publication in peer reviewed medical journal and will be presented at international conferences on infectious diseases in the form of an oral presentation or poster.

**Ethical considerations**

**Protection of human subjects**

**Consent**

Delayed informed consent is planned for this study to enable the rapid onset of the bundle delivery, and may be considered due to the following:

**Standard informed consent processes are not appropriate for testing emergency interventions**

GCP informed consent can only be given when the subject is physically and emotionally well enough to receive and comprehend the details given to the subject in the information sheet and by the study worker. Due to the high likelihood of comprehension being impaired during an acute episode of ABM, it is not appropriate to take informed consent at the acute admission point. Once the subject has been stabilised, and has improved to a point where s/he has reasonable comprehension and retention of information, formal written informed consent will be requested. This is known as deferred consent. However the bundle will need to be started before the patient is stabilised, which is standard recognised practice in studies of emergency interventions.

**No new drug or treatment is being tested**

The bundle consists of normal, recognised treatments, each with evidence of efficacy that do not require informed consent to administer in standard clinical practice.

**Risk of delays in informed consent affecting the study outcome**

The aim of the bundle is speed of onset, and delays inherent in the informed consent process have the potential to impact on the success of the treatment.

**Previous published experience in deferred consent**

The approach of delayed formal informed consent has been well validated internationally in the conduct of all clinical trials where rapidity of intervention onset is a major priority for efficacy, as per FDA guidelines in 1996 (40). Initial discussion followed by delayed informed consent is commonly used in this setting and is accepted practice (41) and is currently being used in the FEAST trial ([www.feast-trial.org](http://www.feast-trial.org)), a large MRC funded multicentre study of acute fluid resuscitation in sick children in East Africa(42).

For most published studies testing sepsis bundle implementation using a before/after design, the need for informed consent was waived at institutional ethical review (27, 33-37). Those which required informed consent did not discuss the rationale for the consent process in the methodology (43-44). One study identified did not obtain informed consent from participants, and did not discuss any ethical concerns, or disclose institutional ethical review (38).

**Confidentiality**

**Confidential data**

All data will be treated as confidential. Data entry will use only the barcode from the Spine system as identifying information and no personal data that could identify the trial participant will be kept within the study data management system. The Spine system is an electronic patient record that is started by the admitting nurse for each patient attending QECH. It contains daily updated information from the inpatient team including diagnosis, medication, discharge dates or cause of death. It is accessible through a barcode that is printed on the patient’s hand held record ‘patient passport’, and requires a secure user password for entry to the record. The results of laboratory tests are also due to be released by the MLW lab onto Spine. Therefore by using the same barcode system we have the same level of security to protect our data. A separate written record of the demographic details of each study participant will be kept for the purposes of ensuring follow up information is obtained by mobile telephone. This will be kept in a locked office separately from the clinical database, and no clinical data will be recorded at this location. The study identifiers will be kept with this record. This record will be destroyed at the end of the study.

**Data sharing**

Data will be shared with a limited number of people to preserve confidentiality. All study team members will have access to the data entry points via an individual password. As records will require a barcode to be scanned to locate each record, this will help protect the data on that system. All data once downloaded and stored in the MLW server will be deleted from the portable computer system to improve data security. Weekly downloads of anonymous data will be sent each month by secure email to the study monitor.

Summaries of the data from each phase will be downloaded and presented to the TSC for discussion when necessary. All data will only be shared with trusted members of the study team using encrypted email.

Where scientific opinion or collaboration is considered useful, anonymous data including clinical samples may be shared with potential collaborators. Permission will be sought for this via the informed consent process at entry into the study

**Burden and risks to study participants**

The study participants will benefit from increased nursing care and it is estimated that the risk of harm from additional observations and clinical care is minimal. The elements of the bundle are all designed to improve patient care, and therefore will not impose a significant burden on the study subjects. The independent study monitor will oversee all monthly mortality figures and SAEs to monitor for any potential harm. Potential risks to the study participants include the following. If any occur a SAE/SUE will be triggered.

| **Potential risk** | **Remedial action** |
| --- | --- |
| Excessive administration of intravenous fluids leading to cerebral oedema | Careful monitoring, administration of diuretics, head elevation, further staff training and supervision |
| Excessive administration of anti-seizure therapy to control a short fit leading to respiratory depression | Airway management, oxygen, ventilation if necessary, further staff training and supervision |
| Damage to the lining of the nose during insertion of airway support when in coma | Removal of the airway and re-insertion with a smaller tube in the alternative nostril |
| Transfusion reaction during blood transfusion | Transfusion will be stopped and intravenous fluid and steroids will be given. Blood bank to be informed and given the transfusion details |
| Transmission of blood borne viruses during blood transfusion | Participant or carer to be counselled regarding the risks and benefits of transfusion. No transfusion will be given without consent. |

**Before/After study design**

As each element of the bundle intervention planned for this study has supportive evidence of efficacy, it is unethical to randomise subjects to receive placebo in a traditional parallel design. It is essential however to have a prospective control population against which to judge the efficacy of the intervention hence the before/after design. As the level of clinical care provided in the AETC is not yet known, but is assumed to be better than that currently available on the medical ward. Prospective data must be collected to inform the baseline mortality rates and clinical care levels.

Ethical issues may be raised by the study team caring for the control subjects in Phase 1 if due to understanding of the clinical evidence that informs the care bundle, they feel more treatment should be given than is currently prescribed for an individual subject. Support will be given by the PI where necessary to discuss and resolve any ethical concerns that may arise from the study team.

**Sustainability**

To facilitate recruitment over the study period, this study has a far higher nurse: patient ratio than is traditionally seen in hospitals in resource limited countries, and the bundle contains elements which may not be available in every setting. However as the bundle concept is a test of proof of principle, it is important to test it with the best and most rigorous methodology possible, to create the highest possible standard ‘gold standard’ of patient management. If successful, bundle elements appropriate to smaller, district general hospitals may be tested in a larger, cluster randomised design with lower nurse: patient ratios, the results of which would be more applicable to general resource limited settings.

The nursing levels within BAM are not currently sustainable outside of the study setting. However the teaching and support provided by the study team during the 2 years to the AETC nurses and doctors will provide a valuable training resource to ensure that all sick patients are seen promptly and given effective management. The study team will provide regular feedback on the study progress within the AETC, and donate all remaining equipment at the end of the study to the department, including two oxygen concentrators. While in the department, the study nurses will assist with other patients when they are not busy with study subjects, subject to a written agreement. If the study demonstrates a mortality benefit from the bundle, the study team will write guidelines for the AETC on the management of suspected ABM for use by all QEH and College of Medicine staff. All treatment elements of the bundle are sustainable in a hospital with basic medical facilities and can be administered by all grades of medical staff.

**Storage and further use of clinical samples**

The pathogenesis of adult bacterial meningitis and pneumococcal meningitis in general is a poorly understood and most current knowledge comes from animal models of infection. These may not be representative of human disease, particularly in immune-suppressed adults in resource limited countries, for which there is minimal current research and a huge clinical problem. CSF and serum samples removed with permission following informed consent have great potential to be analysed to increase our understanding of ABM in this setting.

I have planned that all future study work involving samples retained from this study will have aims towards translatable results into clinical medicine (i.e. search for biomarkers of severity) and those which may in the longer term inform vaccine and treatment adjunct design. All samples will not be used for any other purposes apart from those stated in section iv.v; methodology, on page 17 and this is explicit in the study information sheet. All retained samples and associated data will only be shared with the collaborators listed on this protocol.

We plan only to request informed consent to retain samples that are surplus to diagnostic requirements and would be removed from the subject in a diagnostic sample set and only the remainder retained. Therefore no additional procedures would be required to obtain samples for further research work.

**Possible constraints**

This study will use 4 nurses and one clinical officer, entirely funded by the study budget. Therefore there will be no loss of staff from patient care. The study nurses and clinical officer are likely to have a large capacity benefit to the AETC as they will care for a significant number of sick new admissions, reducing the burden on the nurses and doctors. All equipment, drugs and intravenous fluids will also be supplied by the study, and all unused equipment will be donated to the department at completion. No resources will be lost from the AETC as a result of hosting the study. Basic diagnostic tests will be performed in the MLW laboratory, which will be equivalent to the normal work-load, and therefore should place only minimal extra stress on the staff and resources in the lab. Outpatient follow up will be in a dedicated facility and will not detract from the limited space available for clinical care.

**Low recruitment numbers**

It is possible due to the roll out of antiretroviral medication for HIV (ART) that the numbers of subjects with ABM may be lower than seen previously, as declining numbers of all pneumococcal infections have been documented in the last two years. This study will recruit all possible subjects, in equal numbers in each group and will provide useful data, independently of the numbers recruited.

**Systematic problems within the AETC**

As the AETC is not yet functional, it is impossible to predict if this study will cause significant systematic problems within the AETC. The study subjects, once recruited, will need to remain in the AETC for 6 hours under observation. This may take space required for newer urgent cases. If this is a systematic problem, discussions will occur between the PI and the clinical director of the AETC to ensure a safe resolution.

**Requirements**

**Personnel**

This study requires 4 study nurses working in a shift system to cover recruitment and follow up between 8am and 11pm, 7 days per week. One clinical officer is required to perform clinical assessments of all potential study participants, perform lumbar puncture and prescribe all relevant medication in Phase 2.

**Capacity strengthening**

This project forms part of the core research and training activities of the Malawi-Liverpool-Wellcome Programme and will be one of the first studies to provide clinical and research infrastructural support to the new AETC. This study will provide valuable experience for a local clinical and laboratory staff members within MLW with an emphasis on study design, analysis and critical appraisal; ethics; and good clinical practice.

This study has the potential to improve clinical care delivery, over time to all sick patients presenting to QECH. By assessing the feasibility of EGDT in this setting, it is hoped that in the longer term a system of ETAT, similar to that in paediatrics can be developed.

All study team members will undergo significant training and support over the 2 years of the study and will gain increased clinical and research experience. The study staff will care for a significant number of acutely unwell patients in the AETC, relieving the burden on the other staff. They will also, subject to agreement within the AETC, assist other nurses with the care of patients when they are not occupied with study patients.

The presence of the study in the AETC will enable ongoing training and support for all members of staff in the management of sick patients, particularly those with brain infections. Regular teaching and feedback seminars will be held by the study team to interact with the AETC staff and allow for open discussion about the impact of the study. The study team will write guidelines for the management of ABM, and it is hoped that a short training course in resuscitation can be developed by the study nurses to enable ongoing training and support for nurses and doctors working in acute medicine. All remaining study equipment will be donated to the AETC at the end of the study.

**Training**

All study members will receive GCP training in research practice and informed consent, funded from the study budget. This, combined with the ongoing training throughout the study will significantly increase their research and clinical experience and will assist with career progression.

**Transport**

Study subjects will be asked to come back to outpatient follow up at 6 weeks from discharge. Expenses will be paid for this. Where a subject is unable to attend follow up for personal, geographical or financial reasons, attempts to collect data over the telephone will be made.

Staff leaving work at the end of the late shift will be driven home by taxi, funded from the study budget to enable secure and safe travel after hours.

**Other requirements**

Laboratory space and the relevant machines for performing real-time PCR are available within the MLW laboratory.

**Budgetary estimates**

All funding for salaries, consumables and associated research expenses are covered by a research grant from the Wellcome Trust to Dr Emma Wall CNR 11145 as part of the Liverpool- Wellcome Trust Clinical PhD programme. ***The budget is in UK pounds sterling.***

| **Research expenses** | **Costs** |
| --- | --- |
| ***Salaries*** |  |
| Senior study nurse (x1) 2years | 16848.3 |
| Junior study nurse (x3) 2 years | 37881 |
| Clinical Officer (x1) 2 years | 16848.3 |
| **Salaries Subtotal** | **71577.6** |
| ***Direct project costs*** |  |
| Project staff related costs (uniforms/registration etc) | 4580 |
| Staff transport (after late shift at 11pm daily) | 5840 |
| Clinical equipment (needles/syringes/oxygen tubing/gloves etc.) | 6897.3 |
| Clinical laboratory tests (FBC/Sodium/Creatinine/HIV) | 15993.3 |
| Real-Time PCR consumables (DNA extraction and amplification consumables) | 3893.3 |
| Patient telephone contact and transport | 1800 |
| Office related costs (printer, stationary, photocopying, IT support) | 3300 |
| Shipping and Clearing | 15000 |
| Insurance | 90 |
| Translation | 300 |
| Data entry and statistics | 5000 |
| Health and safety (including PEP) | 1500 |
| Ethics and poisons board fees | 2460 |
| **Project costs subtotal** | **66654** |
| MLW support/administration costs | 14600 |
| Travel and subsistence subtotal | 10500 |
| **Subtotal** | **163331** |

**Justification of the budget**

This study has a large budget over 3 years. This is predominately needed to fund adequate levels of staffing to ensure maximal recruitment and appropriate delivery of the intervention.

**Staffing**

If study staff were only available during normal working hours, recruitment will be limited to < 30% of the numbers required and many patients at highest risk would not be included, severely limiting the assessment of the bundle. It is therefore essential that the study team be available to recruit and deliver the bundle beyond the standard 8am-5pm working day.

To achieve these aims, we need to employ four nurses and one clinical officer over two years. With this team, the study nurses can recruit actively from 7am to 5pm, and complete bundle delivery by 11pm. The planned staffing system is necessary to meet these requirements.

**Clinical equipment**

Each subject will need to be observed for 6 hours and require basic interventions and laboratory investigations. Support is also required to supply all medical equipment required for investigations and to deliver the bundle, as it would be unethical to use limited existing resources for this study.

**Laboratory investigations**

As the exclusion criteria are based on CSF findings, a large number of subjects may be screened and require investigations, only to be subsequently excluded. As many as possible of these tests are being done at the bedside to limit the cost, but costs of essential diagnostic tests are included in the budget.

**Summary**

This is the first time a study of this type has been conducted in an adult resource limited setting and the intervention is unusual in its delivery and duration. The output of this study, if conducted appropriately with an adequate sample size, has the potential to inform important changes to adult meningitis management in resource limited settings, and build capacity within the new AETC in QECH.

**References**

1. Scarborough M, Gordon SB, Whitty CJ, French N, Njalale Y, Chitani A, et al. Corticosteroids for bacterial meningitis in adults in sub-Saharan Africa. N Engl J Med. 2007 Dec 13;357(24):2441-50.

2. Manga NM, Ndour CT, Diop SA, Ka-Sall R, Dia NM, Seydi M, et al. [Adult purulent meningitis caused by Streptococcus pneumoniae in Dakar, Senegal]. Med Trop (Mars). 2008 Dec;68(6):625-8.

3. Gordon SB, Walsh AL, Chaponda M, Gordon MA, Soko D, Mbwvinji M, et al. Bacterial meningitis in Malawian adults: pneumococcal disease is common, severe, and seasonal. Clin Infect Dis. 2000 Jul;31(1):53-7.

4. Rivers E, Nguyen B, Havstad S, Ressler J, Muzzin A, Knoblich B, et al. Early goal-directed therapy in the treatment of severe sepsis and septic shock. N Engl J Med. 2001 Nov 8;345(19):1368-77.

5. Barochia AV, Cui X, Vitberg D, Suffredini AF, O'Grady NP, Banks SM, et al. Bundled care for septic shock: an analysis of clinical trials. Crit Care Med. 2010 Feb;38(2):668-78.

6. Dellinger RP, Levy MM, Carlet JM, Bion J, Parker MM, Jaeschke R, et al. Surviving Sepsis Campaign: international guidelines for management of severe sepsis and septic shock: 2008. Intensive Care Med. 2008 Jan;34(1):17-60.

7. Marwick C, Davey P. Care bundles: the holy grail of infectious risk management in hospital? Curr Opin Infect Dis. 2009 Aug;22(4):364-9.

8. Molyneux E, Ahmad S, Robertson A. Improved triage and emergency care for children reduces inpatient mortality in a resource-constrained setting. Bull World Health Organ. 2006 Apr;84(4):314-9.

9. WHO. Intergrated management of adolescent and adult illnesses. WHO online publications [serial on the Internet]. 2004: Available from: <http://dosei.who.int/uhtbin/cgisirsi/XmxkjFVTqd/36200016/5/0>.

10. Carrol ED, Mankhambo LA, Corless C, Guiver M. Bacteremia is associated with a worse outcome in pneumococcal meningitis. J Infect Dis. 2008 Aug 15;198(4):626-7.

11. Rello J, Lisboa T, Lujan M, Gallego M, Kee C, Kay I, et al. Severity of pneumococcal pneumonia associated with genomic bacterial load. Chest. 2009 Sep;136(3):832-40.

12. The Brain Trauma Foundation. The American Association of Neurological Surgeons. The Joint Section on Neurotrauma and Critical Care. Initial management. J Neurotrauma. 2000 Jun-Jul;17(6-7):463-9.

13. Roberts K, Whalley H, Bleetman A. The nasopharyngeal airway: dispelling myths and establishing the facts. Emerg Med J. 2005 Jun;22(6):394-6.

14. Molyneux AJ. Changes in the treatment of patients with subarachnoid haemorrhage following publication of the International Subarachnoid Aneurysm Trial. Clinical Neurology & Neurosurgery. 2006 Feb;108(2):115-6.

15. Harnden A, Ninis N, Thompson M, Perera R, Levin M, Mant D, et al. Parenteral penicillin for children with meningococcal disease before hospital admission: case-control study. BMJ. 2006 Jun 3;332(7553):1295-8.

16. Auburtin M, Wolff M, Charpentier J, Varon E, Le Tulzo Y, Girault C, et al. Detrimental role of delayed antibiotic administration and penicillin-nonsusceptible strains in adult intensive care unit patients with pneumococcal meningitis: the PNEUMOREA prospective multicenter study. Crit Care Med. 2006 Nov;34(11):2758-65.

17. Durand ML, Calderwood SB, Weber DJ, Miller SI, Southwick FS, Caviness VS, Jr., et al. Acute bacterial meningitis in adults. A review of 493 episodes. N Engl J Med. 1993 Jan 7;328(1):21-8.

18. Maconochie I, Baumer H, Stewart ME. Fluid therapy for acute bacterial meningitis. Cochrane Database Syst Rev. 2008(1):CD004786.

19. Shander A. Anemia in the critically ill. Crit Care Clin. 2004 Apr;20(2):159-78.

20. WHO. The clinical use of blood handbook. Blood transfusion safety [serial on the Internet]. 2009 [cited 2010: Available from: <http://www.who.int/bloodsafety/clinical_use/en/>.

21. Campbell M, Fitzpatrick R, Haines A, Kinmonth AL, Sandercock P, Spiegelhalter D, et al. Framework for design and evaluation of complex interventions to improve health. BMJ. 2000 Sep 16;321(7262):694-6.

22. Lipshutz AK, Fee C, Schell H, Campbell L, Taylor J, Sharpe BA, et al. Strategies for success: A PDSA analysis of three QI initiatives in critical care. Jt Comm J Qual Patient Saf. 2008 Aug;34(8):435-44.

23. Levy MM, Pronovost PJ, Dellinger RP, Townsend S, Resar RK, Clemmer TP, et al. Sepsis change bundles: converting guidelines into meaningful change in behavior and clinical outcome. Crit Care Med. 2004 Nov;32(11 Suppl):S595-7.

24. Gabbott D, Smith G, Mitchell S, Colquhoun M, Nolan J, Soar J, et al. Cardiopulmonary resuscitation standards for clinical practice and training in the UK. Accid Emerg Nurs. 2005 Jul;13(3):171-9.

25. Heyderman RS. Early management of suspected bacterial meningitis and meningococcal septicaemia in immunocompetent adults--second edition. J Infect. 2005 Jun;50(5):373-4.

26. Carrol ED, Guiver M, Nkhoma S, Mankhambo LA, Marsh J, Balmer P, et al. High pneumococcal DNA loads are associated with mortality in Malawian children with invasive pneumococcal disease. Pediatr Infect Dis J. 2007 May;26(5):416-22.

27. Jones AE, Focht A, Horton JM, Kline JA. Prospective external validation of the clinical effectiveness of an emergency department-based early goal-directed therapy protocol for severe sepsis and septic shock. Chest. 2007 Aug;132(2):425-32.

28. Schut ES, Westendorp WF, de Gans J, Kruyt ND, Spanjaard L, Reitsma JB, et al. Hyperglycemia in bacterial meningitis: a prospective cohort study. BMC Infect Dis. 2009;9:57.

29. Brouwer MC, van de Beek D, Heckenberg SG, Spanjaard L, de Gans J. Hyponatraemia in adults with community-acquired bacterial meningitis. QJM. 2007 Jan;100(1):37-40.

30. Dellinger RP, Levy MM, Carlet JM, Bion J, Parker MM, Jaeschke R, et al. Surviving Sepsis Campaign: international guidelines for management of severe sepsis and septic shock: 2008. Crit Care Med. 2008 Jan;36(1):296-327.

31. Goonetilleke UR, Scarborough M, Ward SA, Gordon SB. Proteomic analysis of cerebrospinal fluid in pneumococcal meningitis reveals potential biomarkers associated with survival. J Infect Dis. 2010 Aug 15;202(4):542-50.

32. Sonia Davila VJW, Chiea Chuen Khor,Kar Seng Sim,Alexander Binder,Willemijn B Breunis,David Inwald,Simon Nadel,Helen Betts,Enitan D Carrol,Ronald de Groot,Peter W M Hermans,Jan Hazelzet,Marieke Emonts,Chui Chin Lim,Taco W Kuijpers,Federico Martinon-Torres,Antonio Salas,Werner Zenz,Michael Levin& Martin L Hibberd. Genome-wide association study identifies variants in the CFH region associated with host susceptibility to meningococcal disease. Nature Genetics. 2010;Online publication.

33. Trzeciak S, Dellinger RP, Abate NL, Cowan RM, Stauss M, Kilgannon JH, et al. Translating research to clinical practice: a 1-year experience with implementing early goal-directed therapy for septic shock in the emergency department. Chest. 2006 Feb;129(2):225-32.

34. Castellanos-Ortega A, Suberviola B, Garcia-Astudillo LA, Holanda MS, Ortiz F, Llorca J, et al. Impact of the Surviving Sepsis Campaign protocols on hospital length of stay and mortality in septic shock patients: results of a three-year follow-up quasi-experimental study. Crit Care Med. 2010 Apr;38(4):1036-43.

35. El Solh AA, Akinnusi ME, Alsawalha LN, Pineda LA. Outcome of septic shock in older adults after implementation of the sepsis "bundle". J Am Geriatr Soc. 2008 Feb;56(2):272-8.

36. Kortgen A, Niederprum P, Bauer M. Implementation of an evidence-based "standard operating procedure" and outcome in septic shock. Crit Care Med. 2006 Apr;34(4):943-9.

37. Shapiro NI, Howell MD, Talmor D, Lahey D, Ngo L, Buras J, et al. Implementation and outcomes of the Multiple Urgent Sepsis Therapies (MUST) protocol. Crit Care Med. 2006 Apr;34(4):1025-32.

38. Otero RM, Nguyen HB, Huang DT, Gaieski DF, Goyal M, Gunnerson KJ, et al. Early goal-directed therapy in severe sepsis and septic shock revisited: concepts, controversies, and contemporary findings. Chest. 2006 Nov;130(5):1579-95.

39. Schulz KF, Chalmers I, Hayes RJ, Altman DG. Empirical evidence of bias. Dimensions of methodological quality associated with estimates of treatment effects in controlled trials. JAMA. 1995 Feb 1;273(5):408-12.

40. consent Pohsi. Final rule. Federal Register. 1996;61(192):51498-533.

41. Lemaire F. Emergency research: only possible if consent is waived? Curr Opin Crit Care. 2007 Apr;13(2):122-5.

42. Maitland K, Kiguli S, Opoka RO, Engoru C, Olupot-Olupot P, Akech SO, et al. Mortality after fluid bolus in African children with severe infection. N Engl J Med. 2011 Jun 30;364(26):2483-95.

43. Robb E, Jarman B, Suntharalingam G, Higgens C, Tennant R, Elcock K. Using care bundles to reduce in-hospital mortality: quantitative survey. BMJ. 2010;340:c1234.

44. Micek ST, Roubinian N, Heuring T, Bode M, Williams J, Harrison C, et al. Before-after study of a standardized hospital order set for the management of septic shock. Crit Care Med. 2006 Nov;34(11):2707-13.

**Appendices**

1. Study information sheet (English) Phases 1 &2
2. Study information sheet (Chichewa) Phases 1&2
3. Verbal assent form in English and Chichewa
4. Written consent form (English)
5. Written consent form (Chichewa)
6. Letter of support from the departmental head (Medicine)
7. Letters of support from PhD supervisor Professor R Heyderman, Professor D Lalloo at the WTTC in Liverpool and from the Wellcome Trust.
8. Flowchart of the study design
9. CSF management algorithm
10. Material Transfer Agreement for the transport of RNA samples to Imperial College, UK
