# Supplementary material for: Goal directed therapy for suspected acute bacterial meningitis in adults and adolescents in sub-Saharan Africa
Source: PLoS One. 2017 Oct 27;12(10):e0186687. doi: 10.1371/journal.pone.0186687 (PMC5659601; doi:10.1371/journal.pone.0186687)
Supplement: S1 Table — (DOCX) [file pone.0186687.s003.docx]

| Supplementary Table 1: Physiological and laboratory characteristics on admission of all screened patients with suspected bacterial meningitis | | | |
| --- | --- | --- | --- |
| Characteristic on presentation | **Value or Median value**  **(% or Inter Quartile Range IQR)** | | **Univariate significance of differences between two phases** |
|  | **P1** | **P2** |  |
| Median Glasgow Coma Score | 14 (13-15) | 14 (13-15) | 0.20 |
| GCS >8-≤11 | 24/269 (9%) | 35/290 (12%) | 0.27 |
| GCS ≤8 | 26/269 (10%) | 20/290 (7%) | 0.28 |
| Median Mean arterial blood pressure (mmHg) | 90 (77-100) | 89 (77 – 100) | 0.71 |
| Median Pulse (bpm) | 100 (80-118) | 98 (82-118) | 0.83 |
| Median Capillary refill time (seconds) | 1 (1-2) | 1 (1-2) | 0.002 |
| Median Temperature (°C) | 37.7 (36.6 – 38.7) | 38.0 (36-38) | 0.26 |
| Median Oxygen saturations (%) | 97 (95 – 98) | 97 (95-98) | 0.99 |
| Median Respiratory rate | 24 (20-30) | 21 (20-26) | <0.001 |
| Median Estimated body mass index (BMI)* | 22.2 (20.4 – 23.9) | 22.2 (19.7-23.4) | 0.24 |
| *Laboratory results* |  |  |  |
| Positive blood culture | 82/271 (30%) | 55/285 (19%) | 0.03 |
| Haemoglobin (g/dL) | 11.9 (9.6 – 13.6) | 11.5 (9.1-12.9) | 0.058 |
| White cell count (cells/mm^3^) | 6.5 (4.2 – 10.6) | 6.6 (4.3-10.8) | 0.87 |
| Platelet count (x10^9^/L) | 211 (127 – 293) | 202 (121-289) | 0.83 |
| CD4 count (cells/mm^3^) | 97 (46 – 215) | 98 (72-176) | 0.75 |
| Positive test for *P. Falciparum* Ag | 15/194 (7%) | 17/290 (6%) | 0.51 |
| Blood Glucose (mmol/L) | 6.1 (5.3 – 7.4) | 6.5 (5.5-7.7) | 0.06 |
| Blood lactate (mmol/L) | 2.4 (1.7 – 4.0) | 2.7 (1.7-4.3) | 0.29 |
| Creatinine (μmol/L) | 87.5 (77 – 99) | 79 (62-115) | 0.65 |
| Sodium (mmol/L) | 141 (138 – 143) | 139 (133-144) | 0.82 |

*BMI estimated as most patients unable to stand for weight on admission in AETC. Where recorded, last weight in the health passport used.
