# Supplementary material for: Goal directed therapy for suspected acute bacterial meningitis in adults and adolescents in sub-Saharan Africa
Source: PLoS One. 2017 Oct 27;12(10):e0186687. doi: 10.1371/journal.pone.0186687 (PMC5659601; doi:10.1371/journal.pone.0186687)
Supplement: S2 Table — (DOCX) [file pone.0186687.s004.docx]

**Supplementary Table 2: Final diagnoses of all screened patients with suspected bacterial meningitis**

| Diagnosis | Phase 1  n= 273 | Phase 2  n=290 |
| --- | --- | --- |
| Proven or probable bacterial meningitis (%)* | 71 (26) | 61 (21) |
| Possible bacterial meningitis (%)† | 10 (3) | 17 (6) |
| Cryptococcal meningitis (%) | 36 (13) | 47 (16) |
| TB meningitis (%) | 10 (3) | 28 (9) |
| Suspected Viral meningitis (%) | 0 (0) | 4 (1) |
| Cerebral/ severe malaria (%) | 19 (7) | 8 (3) |
| Bacterial sepsis and no meningitis (%) | 82 (30) | 98 (34) |
| Pneumonia (%) | 8 (3) | 6 (2) |
| Head injury/cerebral haemorrhage/ stroke (%) | 5 (2) | 2 (0.5) |
| Space occupying lesion (%) | 2 (0.7) | 1 (0.3) |
| Withdrew consent (%) | 0 (0) | 3 (1) |
| Other (%) | 30 (10) | 15 (5) |

* positive CSF gram’s stain, culture or retrospective PCR for causative organisms, irrespective of other CSF findings), or probable (negative microbiology with acute history and CSF pleocytosis of >50 cells/mm3 or clumped cells with >50% neutrophils or >50% lymphocytes with prior antibiotics and biochemical evidence of meningitis, CSF: Blood glucose ratio of <0.4, raised CSF protein >0.5g/L)

**†**clinical syndrome of bacterial meningitis, CSF not meeting inclusion criteria with no alternative diagnosis.
