# Supplementary material for: Goal directed therapy for suspected acute bacterial meningitis in adults and adolescents in sub-Saharan Africa
Source: PLoS One. 2017 Oct 27;12(10):e0186687. doi: 10.1371/journal.pone.0186687 (PMC5659601; doi:10.1371/journal.pone.0186687)
Supplement: S3 Table — (DOCX) [file pone.0186687.s005.docx]

| Supplementary Table 3: Summary of CSF and blood culture results for all screened participants with suspected bacterial meningitis | | | | |
| --- | --- | --- | --- | --- |
| Blood culture results | | | **CSF culture &PCR results** | |
|  | **P1** | **P2** | **P1** | **P2** |
| No growth | 167 | 230 | 194 | 211 |
| *Cryptococcus neoformans* | 15 | 21 | 27 | 32 |
| *Streptococcus pneumoniae* | 20 | 3 | 39 | 45 |
| *N.meningitidis* | 0 | 3 | 4 | 7 |
| *Salmonella typhi* | 7 | 3 | 0 | 0 |
| Non-typhoidal *S*almonellae | 7 | 4 | 0 | 0 |
| *E.coli* | 3 | 2 | 1 | 1 |
| Others* | 4 | 6 | 5 | 8 |
| Contaminant† | 26 | 15 | 9 | 7 |

*Includes Group A streptotococci, and *Staphylococcus* aureus

†Predominately coagulase negative staphylococcal species, micrococcus luteus and other skin flora
