# Supplementary material for: Goal directed therapy for suspected acute bacterial meningitis in adults and adolescents in sub-Saharan Africa
Source: PLoS One. 2017 Oct 27;12(10):e0186687. doi: 10.1371/journal.pone.0186687 (PMC5659601; doi:10.1371/journal.pone.0186687)
Supplement: S4 Table — (DOCX) [file pone.0186687.s006.docx]

| Supplementary Table 4: Baseline characteristics of study participants with proven or probable bacterial meningitis | | | |
| --- | --- | --- | --- |
| Characteristic on presentation | **Value or Median value**  **(% or Inter Quartile Range IQR)** | | **Univariate significance of differences between two phases** |
| *Clinical observations* | Phase 1  N=71 | Phase 2  N=61 | P value |
| Female | 30 (42%) | 21 (34%) | 0.37† |
| Median age (years) | 32 (25 – 42) | 34 (27.5 – 43.5) | 0.14† |
| HIV positive | 42/60 (70%) | 40/52 (77%) | 0.65† |
| Clinically defined WHO HIV stage 3/4 | 40 (56%) | 24 (39%) | 0.059† |
| History of WHO stage 3 or 4 condition | 30 (42%) | 27 (44%) | 0.86 |
| Antiretroviral therapy | 20/60 (33%) | 14/57 (24.5%) | 0.20† |
| Out of hours BAM team admission | 23 (32%) | 1 (1.6%) | <0.001† |
| Median Pre-hospital symptom duration (hours) (IQR) | 48 (48 – 72) | 48 (24 – 72) | 0.005 § |
| <24 hrs | 13 | 27 | Ref |
| 24-48 hrs | 25 | 20 | 0.35 |
| 48-72 hrs | 21 | 12 | 0.009 |
| 72-96 hrs | 5 | 1 | 0.041 |
| >96 hrs | 7 | 0 | 0.99 |
| Pre-hospital antibiotics | 35/63 (55%) | 34/57 (60%) | 0.58† |
| History of acute headache | 68 (96%) | 56 (93%) | 0.40† |
| Pre-hospital seizures | 20 (28%) | 9 (14.8%) | 0.049† |
| *Clinical features on presentation* |  |  |  |
| Neck stiffness | 52 (73%) | 42 (68%) | 0.35† |
| Photophobia | 9 (12%) | 17 (27%) | 0.024† |
| Cranial nerve palsy | 1 (1%) | 3 (4.9%)  (III = 2 VIII = 1) | 0.27† |
| Moribund on admission | 11 (15.4%) | 4 (6.6%) | 0.08† |
| Clinical evidence of shock* | 45 | 41 | 0.85† |
| Focal limb weakness | 5 (7%) | 2 (3%) | 0.29† |
| Pre-illness modified Rankin Score >2 | 2 (3%) | 4 (6.5%) | 0.53† |
| Acute seizures in AETC | 7/62 (11%) | 7/58 (12%) | 0.55† |
| Acute presenting physical parameters |  |  |  |
| Glasgow Coma Score | 13 (10 – 14) | 13 (11 - 14) | 0.80† |
| GCS >8-<11 | 12 (16%) | 10 (16.4%) | 0.63≠ |
| GCS <8 | 14 (20%) | 6 (9.8%) | 0.09≠ |
| Median mean arterial blood pressure (mmHg) | 90 (75 – 105) | 92 (82 – 104) | 0.59 § |
| Pulse (bpm) | 101 (85 – 116) | 99 (86 – 119) | 1.0 § |
| Capillary refill time (seconds) | 1 (1-2) | 1 (1-2) | 0.66 § |
| Temperature (°C) recorded | 38.2 (37.1 – 39.1) | 38 (36 – 39) | 0.01 § |
| Oxygen saturations % | 96 (94 – 98) | 97 (94 – 97) | 0.042§ |
| Respiratory rate | 26 (22 - 32) | 21 (19 – 26) | 0.007 § |
| Estimated body mass index (BMI) | 22.4 (20.9 – 24.1) | 23 (21.1 – 24.3) | 0.59 § |
| MAMS (IQR) n=39 | 157 (132 - 182) | 149 (110 - 190) | 0.61 |
| Laboratory results |  |  |  |
| *Microbiology* | **Phase 1**  **N=71** | **Phase 2**  **N=61** | **P** |
| CSF culture | No growth 23 (32%) | 27 (44%) | 0.057 ≠ |
|  | *S.pneumoniae* 34 (47%) | 27 (44%) | 0.11 |
|  | *N. meningitidis* 3 (4.2%) | 1 (1.6%) | 0.59 |
|  | *E. coli* 1 (1.4%) | 1 (1.6%) | 0.27 |
|  | Other 3 (4.2%) | 4 (6.6%) | 0.08 |
|  | Contaminant 7 (10%) | 1 (1.6%) | Reference |
| CSF white cell count (cells/mm^3^) | 68  (16 – 288) | 241  (8.5 – 1116) | 0.64† |
| CSF protein (g/L) | 2.84 (1.8 – 5.4) | 2.73 (1.85 – 4.16) | 0.61 § |
| CSF : Blood glucose ratio | 0.34 (0.10 – 0.46) | 0.13 (0.01 – 0.16) | 0.04 § |
| CSF lactate (mmol/L) | 9.8 (7.8 – 11.1) | 10.0 (9.1 – 11.2) | 0.42 § |
| Positive blood culture | 20 (28%)  (18/20) | 8 (13%)  (3/8) | 0.06 ≠ |
| *Blood parameters* |  |  |  |
| Haemoglobin (g/dL) | 11.9 (10.3 – 13.3) | 11.6 (9.9 – 13) | 0.85 § |
| White cell count | 9.6 (6.3 – 14.4) | 10.2 (5.8 – 17.5) | 0.56 § |
| Platelet count | 228 (129 – 315) | 206 (148 – 299) | 0.92 § |
| CD4 count | 97 (41 – 293) | 131 (76 – 249) | 0.94 § |
| Positive test for *P. Falciparum* Ag | 5/53 (9.4%) | 3/61 (4.9%) | 0.46 † |
| Glucose (mmol/L) | 7.1 (5.6 – 8.6) | 7.4 (6.0 – 9.2) | 0.56 § |
| Blood lactate (mmol/L) | 2.9 (2.2 – 5.6) | 3.4 (1.9 – 5.75) | 0.65 § |
| Creatinine (mg/dL) | 0.99 (0.73 – 1.07) | 0.9 (0.72 – 1.30) | 0.89 § |
| Sodium (mmol/L) | 140 (138 – 144) | 139 (133 – 149) | 0.95 § |
