# Supplementary material for: Goal directed therapy for suspected acute bacterial meningitis in adults and adolescents in sub-Saharan Africa
Source: PLoS One. 2017 Oct 27;12(10):e0186687. doi: 10.1371/journal.pone.0186687 (PMC5659601; doi:10.1371/journal.pone.0186687)
Supplement: S5 Table — (DOCX) [file pone.0186687.s007.docx]

| Supplementary Table 5: Composite achievement of clinical targets between Phase 1 and 2 for proven or probable bacterial meningitis | | | | |
| --- | --- | --- | --- | --- |
| Number of targets set | **Phase 1**  **Number of targets achieved/set (%)**  **N=71** | **Phase 2**  **Number of targets achieved/set (%)**  **N=61** |  | **P** |
| 0* | 19/0 | 10/0 |  |  |
| 1† | 15/16 | 18/16 |  | 0.03 |
| 2 | 11/22 | 22/20 |  | 0.66 |
| 3 | 6/14 | 10/11 |  | 0.59 |
| 4 | 0/13 | 1/8 |  | 0.64 |
| 5 | 0/6 | 0/3 |  | **NA** |
| 6 | 0/0 | 0/3 |  | **NA** |
| 7 | 0/0 | 0/0 |  | **NA** |
|  |  |  | **Rate ratio**  **(95% CI)** | **p-value** |
| Mean number of targets set (Std) | 2.59 (1.26) | 2.52 (1.25) |  | **0.77** |
| Mean number of targets achieved (Std) | 1.07 (1.03) | 1.57 (1.00) |  | **0.012** |
