# Supplementary material for: Goal directed therapy for suspected acute bacterial meningitis in adults and adolescents in sub-Saharan Africa
Source: PLoS One. 2017 Oct 27;12(10):e0186687. doi: 10.1371/journal.pone.0186687 (PMC5659601; doi:10.1371/journal.pone.0186687)
Supplement: S6 Table — (DOCX) [file pone.0186687.s008.docx]

| Supplementary Table 6: Clinical targets achieved by phase for patients with proven or probable bacterial meningitis at the end of 6 hours | | | |  |
| --- | --- | --- | --- | --- |
| Parameter | **Target** | **Phase 1**  **N=71** | **Phase 2**  **N=61** | **Univariate significance** |
| Timing of clinical assessment | Medical review <1 hour of arrival | Median time hh:mm (IQR)  0:26 (0:10 – 1:01) | Median time hh:mm (IQR)  0:22 (0:15 – 0:45) | 0.73 |
| Proportion that met the target |  | Proportion seen in <1 hour n=44/62 (70%) | Proportion seen in <1 hour n=48/57 (84%) | 0.12 |
| Antibiotic therapy timing | 1^st^ dose within 1 hour of arrival | Median time hh:mm (IQR)  1:55 (1:10 – 2:52) | Median time hh:mm (IQR)  1:13 (0:42 – 1:58) | <0.001 |
| Proportion met antibiotic target |  | Proportion IVABx <1 hour  n=6/49 (14%) | Proportion IVABx < 1 hour 27/61 (44%) | <0.001 |
| Brain protection | Airway if GCS <8 given?  Head tilt if GCS <11 given? | n=0/14 (0%)  n=0/26 (0%) | n=7/9 (77%)  n=17/19 (89%) | 0.04  0.002 |
| Oxygenation on admission | Give oxygen if Sp02 <94% | Proportion SpO_2_ <94% on admission  = 18/66 (27.2%) | Proportion SpO_2_ <94% on admission  = 15/61 (24.5%) | 0.84 |
| Oxygenation on discharge |  | Proportion SpO_2_ >94% on discharge to ward  = 30/37 (81%) | Proportion SpO_2_ >94% on discharge to ward  = 48/53 (90.5%) | 0.22 |
| Perfusion status on admission | CRT<2 sec, BP >90 mmHg or MAP >70 mmHg, Pulse <100 bpm, lactate >4mmol/L | Proportion with one or more feature of shock on admission  =45/69 (65%) | Proportion with one or more feature of shock on admission  = 41/61 (67%) | 0.85 |
| Perfusion status on discharge |  | Proportion without shock on ward discharge  =20/37 (54%) | Proportion without shock on ward discharge  =31/53 (58%) | 0.82 |
| Blood transfusion | Haemoglobin <6g/dL  Transfusion in AETC? | 2/39 (5%)  0 (0%) | 3/45 (6%)  2/3 (66%) | 1.0  0.4 |
| Seizures | Control of AETC seizures by ward discharge | 7/7 (100%) | 6/7 (85%) | 1.0 |
| Hypoglycaemia on admission | Blood glucose >4 throughout care bundle | Proportion <4mmol/L  0/53 (0%) | Proportion <4mmol/L  1/61 (1.6%) | 0.71 |
