# Supplementary material for: Goal directed therapy for suspected acute bacterial meningitis in adults and adolescents in sub-Saharan Africa
Source: PLoS One. 2017 Oct 27;12(10):e0186687. doi: 10.1371/journal.pone.0186687 (PMC5659601; doi:10.1371/journal.pone.0186687)
Supplement: S7 Table — (DOCX) [file pone.0186687.s009.docx]

**Supplementary Table 7: Outcome of patients with suspected bacterial meningitis; and patients with proven or probable bacterial meningitis**

| Outcome of patients with suspected bacterial meningitis | Phase 1  No. (%) | Phase 2  No. (%) | Odds Ratio  (95% CI) | p-value |
| --- | --- | --- | --- | --- |
| Death by 6 hours | 21/263 (8) | 30/290 (10) | 2.1 (0.79,1.56) | 0.13 |
| Death by 48 hours | 31/263 (12) | 43/290 (15) | 0.79 (0.47,1.31) | 0.36 |
|  |  |  |  |  |
| Outcome of patients with proven or probable bacterial meningitis |  |  |  |  |
| Death by 6 hours | 4/71 (5) | 7/61 (11) | 2.2 (0.61,7.9) | 0.22 |
| Death by 48 hours | 20/71 (28) | 22/71 (36) | 1.4 (0.6,3.0) | 0.33 |
| Death day 10 | 27/71 (38) | 32/71 (52) | 1.8 (0.89,1.36) | 0.09 |
| Death or disability day 10 | 33/71 (46) | 44/61 (72) | 2.9 (1.4,6.1) | 0.004 |
| Death day 40 | 28/57 (49) | 38/60 (63) | 1.8 (0.85,3.7) | 0.13 |
| Death or disability day 40 | 29/57 (51) | 38/60 (63) | 1.7 (0.79,1.4) | 0.19 |
